# Ammonium fertilization increases the susceptibility to fungal leaf and root pathogens in winter wheat

**DOI:** 10.3389/fpls.2022.946584

**Published:** 2022-09-09

**Authors:** Niels Julian Maywald, Melissa Mang, Nathalie Pahls, Günter Neumann, Uwe Ludewig, Davide Francioli

**Affiliations:** Department of Nutritional Crop Physiology, Institute of Crop Science, University of Hohenheim, Stuttgart, Germany

**Keywords:** disease management, crop-pathogen interactions, plant-microbe interactions, nitrogen, nitrate, cyanamide, *Blumeria graminis*, *Gaeumannomyces graminis*

## Abstract

Nitrogen (N) fertilization is indispensable for high yields in agriculture due to its central role in plant growth and fitness. Different N forms affect plant defense against foliar pathogens and may alter soil–plant-microbe interactions. To date, however, the complex relationships between N forms and host defense are poorly understood. For this purpose, nitrate, ammonium, and cyanamide were compared in greenhouse pot trials with the aim to suppress two important fungal wheat pathogens *Blumeria graminis* f. sp. *tritici* (*Bgt*) and *Gaeumannomyces graminis* f. sp. *tritici* (*Ggt*). Wheat inoculated with the foliar pathogen *Bgt* was comparatively up to 80% less infested when fertilized with nitrate or cyanamide than with ammonium. Likewise, soil inoculation with the fungal pathogen *Ggt* revealed a 38% higher percentage of take-all infected roots in ammonium-fertilized plants. The bacterial rhizosphere microbiome was little affected by the N form, whereas the fungal community composition and structure were shaped by the different N fertilization, as revealed from metabarcoding data. Importantly, we observed a higher abundance of fungal pathogenic taxa in the ammonium-fertilized treatment compared to the other N treatments. Taken together, our findings demonstrated the critical role of fertilized N forms for host–pathogen interactions and wheat rhizosphere microbiome assemblage, which are relevant for plant fitness and performance.

## Introduction

The control of phytopathogenic microorganisms like *Blumeria graminis* f. sp. *tritici* (*Bgt*) and *Gaeumannomyces graminis* f. sp. *tritici* (*Ggt*) is crucial for high quality and yield stability in modern agriculture (Peng et al., 2021). Therefore, pesticides are extensively used worldwide. However, due to pesticide residues in food, in the environment, and because of threats to biodiversity, the use of synthetic chemical pesticides is increasingly criticized, and there are demands to reduce their application (Pogacean and Gavrilescu, 2009; Jacquet et al., 2022). Organic farming represents a cultivation system that does not require synthetic chemical pesticides (Seufert et al., 2017), but the higher land use, lower product quality and reduced food supply compared to conventional farming limit its general application (Niggli et al., 2008). The development of an improved and sustainable farming system suitable to supply the growing world population with sufficient high-quality food is therefore one of the greatest challenges of this century (Giller et al., 2021; Zimmermann et al., 2021).

Mineral fertilization targeted at pathogen defense has been neglected in the recent past due to the high use of chemical pesticides. However, it has been widely shown that the addition of mineral nutrients can alter plant physiology, genetics, and plant-associated microbiota and improve plant resistance to biotic and abiotic stresses (Fernandes and Rossiello, 1995; Walters and Bingham, 2007; Geisseler and Scow, 2014; Ding et al., 2021). In this context, the focus has mostly been on the macronutrient nitrogen (N), whose uptake and utilization is the primary limiting factor for plant growth and crop yield. In most cases, nitrogen fertilization has a negative impact on plant resistance to pathogens (Sun et al., 2020). For instance, increased susceptibility to *Bgt* has been found in cereals with N fertilization (Last, 1953; Jensen and Munk, 1997).

It is known that specific forms of nitrogen can lead to different effects on plant resistance. This is attributed to many factors and is not the same for all host–parasite associations (Mur et al., 2017; Sun et al., 2020). Most of the benefits of a particular form of nitrogen have been observed in moderately susceptible or partially resistant crop varieties (Huber and Watson, 1974). The results of previous experimental studies also suggest that the source of nitrogen may be as important as the rate of application (Baker and Martinson, 1970; Huber and Watson, 1974). The infestation-influencing effect of nitrate and ammonium fertilization depends strongly on the host plant and the emerging pathogen (Sun et al., 2020). In general, it has been observed that nitrate can increase the resistance of the plant in a concentration-dependent manner. Investigations into the mechanisms behind this revealed a stronger hypersensitive response (HR) and higher pathogenesis-related (PR) gene expression (Mur et al., 2017). Elevated levels of salicylic acid (SA) were also found in nitrate fertilized plants. SA is the major pathogen defense hormone in plants against biotrophic and hemibiotrophic pathogens (Fagard et al., 2014). Nitrate also increases the production of polyamines such as spermine and spermidine, which can act as defense signals (Mur et al., 2017). Nitric oxide (NO) production and associated defense mechanisms are also nitrate-dependent. NO has been shown to be released following early basal defenses initiated by pathogen-associated microbial patterns or gene-for-gene mediated defenses (Delledonne et al., 1998; Mur et al., 2005; Chen et al., 2014). Instead, ammonium fertilization leads to reduced plant resistance to pathogens through an increase in apoplastic sugars and amino acids (Gupta et al., 2013). The availability of these N compounds could favor the spread of pathogens. Indeed, reduced HR and lower levels of NO may promote pathogen infestation (Planchet et al., 2005; Gupta et al., 2013).

Cyanamide is a classical nitrogen source that has been used mainly for its herbicidal effect, although its application can also reduce fungal diseases (Verona, 1970). Heitefuss and Brandes (1970) reported that cyanamide reduced *Bgt* infection in wheat compared to ammonium nitrate fertilization under field conditions. Despite these positive effects, cyanamide fertilization is now considered controversial, as harmful effects on humans and the environment cannot be excluded (European Chemicals Agency, 2018).

In this study, we investigated the role of nitrogen sources, including nitrate, ammonium, and cyanamide, on *Bgt* and *Ggt* infestation in winter wheat to gain insights into the plant’s physical and biochemical mechanisms mediated by the interaction between nitrogen source and pathogen. To assess the impact of different N forms on the wheat root-associated microbiota, we characterized the bacterial and fungal community associated with the wheat rhizosphere using next-generation sequencing. We hypothesized that (1) mineral N fertilization in form of ammonium would increase the disease severity of foliar and soil-based fungal pathogens compared to nitrate or cyanamide fertilization, and (2) increased pathogenic susceptibility would be associated with biomass loss. In addition, we hypothesized that (3) microbial composition and structure would be affected differently by the varying forms of mineral N fertilization. The overall objective of this study was to demonstrate that the choice of nitrogen form can prophylactically suppress foliar or soil-borne pathogens, thus providing a promising alternative to chemical pesticides.

## Materials and methods

### Experiments with *Blumeria graminis* f. sp. *tritici*

#### Experimental setup

Winter wheat *Triticum aestivum* cv. Emerick (KWS Saat SE & Co. KGaA, Einbeck, Germany), which is partially resistant to *Bgt,* was maintained for 6 weeks in a greenhouse compartment at 21/18°C with a relative humidity of 70% and a photoperiod of 12 h. Per pot, two wheat plants were cultivated. Each pot contained 2 kg of a topsoil and quartz sand mixture (70:30 w:w) with the soil properties given in Supplementary Table 1. In addition, each pot was fertilized with 80 mg kg^−1^ Ca(H_2_PO_4_)_2_, 150 mg kg^−1^ K_2_SO_4_, and 50 mg kg^−1^ MgSO_4_. The three different nitrogen treatments, nitrate, ammonium, and cyanamide, were-fertilized with 200 mg kg^−1^ of the corresponding nitrogen source using calcium nitrate, ammonium sulfate with nitrification inhibitor (3,4-dimethylpyrazole phosphate) (NovaTec Solub 21, Compo GmbH, Münster, Germany), and calcium cyanamide (Perlka, AlzChem Group AG, Trostberg, Germany). The pots were watered by weight up to a water holding capacity (WHC) of 80% when the water holding capacity fell below 40%.

The experiment consisted of a randomized complete block design with six blocks. A block design was chosen to minimize the effect of a temperature gradient in the greenhouse. Each block contained two replicates of each treatment. After 2 weeks of cultivation, three blocks were inoculated with *Bgt*. The three uninoculated blocks were kept separated from the inoculated blocks to avoid pathogen infestation without pesticides (Supplementary Figure 1A).

#### Inoculum production, inoculation, and disease assessment

To produce the inoculum, 2-week-old wheat plants of the cultivar Akteur were infected by cultivating them with already heavily infected wheat plants in a growth chamber. To ensure a consistently high level of viable inoculum, old conidia were shaken off the plants 24 h before inoculation. The infected plants were then used to inoculate the experimental plants by gently dusting the *Bgt* conidia over the experimental plants. The spore density of about 400 conidia cm^−2^ was estimated by counting the number of spores per unit area on coverslips placed in the dusted area. Plants were inoculated on the 14th day after sowing. The inoculated plants were then observed daily for powdery mildew development. Visual assessment of fungal infestation was done 14, 21, and 28 days post-infection (dpi). Based on the scorings of Trommer (1989) and Bundessortenamt (2000), a scale with 10 disease severity levels ranging from 0 to 9 was established, where 0 = no infestation, 1 = 1% infestation of the leaf area, 2 = 3%, 3 = 5%, 4 = 7.5%, 5 = 10%, 6 = 17.5%, 7 = 25%, 8 = 32.5%, 9 = >40%. Then, the disease index was calculated as described in Huang et al. (2007):


$$DI=\frac{\sum_{n=0}^{n}\left(x\times f\right)}{n\times \sum_{n=0}^{n}f}\times 100\text{,}$$

where *x* is the disease severity degree, *f* the total number of leaves of each degree of disease severity and *n* is the highest observed degree of disease severity.

#### Plant analyses

The dry weight of the above-ground plant tissue was determined after 6 weeks of cultivation. The plant parts were dried at 40°C for 1 week and then weighted. Chlorophyll concentration was estimated at 21, 28, 35, and 42 days after sowing. To determine the chlorophyll concentration in the leaves, reflectometric leaf chlorophyll measurements were performed using a Minolta SPAD-502plus (Konica Minolta INC, Osaka, Japan) by measuring the last fully developed leaf three times. SPAD values were then converted into leaf chlorophyll concentration using the formula developed for wheat in Uddling et al. (2007): [μg/cm^2^] = $5.99\times e^{0.0493\times \mathrm{SPAD}\_\mathrm{Value}}$ .

#### Analysis of mineral nutrients

Total nitrogen, carbon, and sulfur were determined with the Vario MAX CNS elemental analyzer (Elementar Analysensysteme GmbH, Langenselbold, Germany). For the determination of mineral nutrients, 250 mg of dried, ground shoot material was weighed in and ashed 5 h in a muffle furnace at 500°C. Afterward, the samples were dried twice on a hot plate after adding 2.5 ml HNO_3_ (1:3) and then dissolved in 2.5 ml HCl (1:3). The samples were then rinsed with 12.5 ml of hot, deionized water and boiled for at least 2 min after adding 2–3 boiling stones, to convert the meta-and pyrophosphates to orthophosphates. After cooling the samples to room temperature, the solution was filled up to 12.5 ml with deionized water, shaken, and filtered through a blue ribbon filter. To measure the iron and manganese concentrations, 0.1 ml caesium chloride/lanthanum chloride buffer solution was added to 4.9 ml ash solution. For the determination of phosphate, 3 ml vanadate-molybdate reagent was added to the ash solution and filled up with HCl (1:30) to a total volume of 10 ml (Gericke and Kurmies, 1952). The potassium and calcium concentrations were measured by an ELEX 6361 flame emission photometer (Eppendorf, Hamburg, Germany). The concentrations of magnesium, iron, zinc, and manganese were determined with an ATI Unicam Solaar 939 atomic absorption spectrometer (Thermo Electron, Waltham, United States). The phosphate content was determined by measuring the absorbance at 436 nm with a U-3300 spectrophotometer (Hitachi Ltd., Tokyo, Japan).

#### Superoxide dismutase assay

The superoxide dismutase (SOD) assay was performed according to the method described by Beauchamp and Fridovich (1971). 100 mg of frozen plant tissue were ground in liquid nitrogen and extracted with 1.5 ml extraction buffer (25 mM HEPES (pH 7.8), 0.1 mM EDTA). After centrifugation at 10,000×*g* at 4°C for 10 min, 100 μl supernatant was added to 300 μl 62.5 mM HEPES (pH 7.8), 75 μl 1 mM EDTA, 75 μl 20 mM Na_2_CO_3_, 75 μl 120 mM L-methionine, 150 μl 750 μM nitro blue tetrazolium (NBT) and 225 μl 10 μM riboflavin. As a control, 100 μl extraction buffer was added to the reaction mixture instead of 100 μl supernatant. The light reaction was started by exposing the samples at 25°C with 8,000 lux. Subsequently, the absorbance was measured spectrophotometrically at 560 nm using a FLUOstar Omega microplate reader (BMG Labtech, Ortenberg, Germany). The SOD activity inhibiting the NBT reduction was calculated according to the following formula: Units of SOD = $\left[\left(A_{\mathrm{Control}}\_A_{\mathrm{Sample}}\right)÷\left(50\%\;A_{\mathrm{Control}}\right)\right]\text{.}$ The specific SOD activity was then expressed as units of SOD per mg of total protein.

#### Determination of H_2_O_2_

The spectrophotometric determination of the hydrogen peroxide content was carried out according to Velikova et al. (2000). For this purpose, 100 mg of frozen plant tissue was ground to a powder in liquid nitrogen. The frozen powder was homogenized with 2 ml 0.1% trichloroacetic acid (w:v). After centrifugation at 12,000×*g* for 15 min, 0.5 ml of the supernatant were added to 0.5 ml 10 mM potassium phosphate buffer (pH 7.0) and 1 ml 1 M potassium iodide. The absorbance was measured spectrophotometrically at 390 nm using a FLUOstar Omega microplate reader (BMG Labtech, Ortenberg, Germany). The H_2_O_2_ content was calculated using a standard curve in the range of 0–120 μM H_2_O_2_.

#### Determination of total antioxidant capacity

The free radical scavenging activity of natural antioxidants was evaluated according to Brand-Williams et al. (1995). 100 mg of frozen plant tissue was ground to a powder in liquid nitrogen and extracted with 1 ml of ethanol:H_2_O (1:1). After centrifugation at 4°C for 10 min at 10,000×*g*, 28 μl of the supernatant were added to 3 mM 1,1-diphenyl-2-picrylhydrazyl radical (DPPH•) ethanol solution and 944 μl ethanol. The DPPH• solution was stored at 4°C in the dark and covered with aluminum foil. Additionally, a blank was prepared containing 28 μl 3 mM DPPH• solution and 972 μl ethanol. The reaction and blank mixtures were incubated at room temperature in the dark for 10 min. Spectrophotometric determination was performed at 515 nm using a FLUOstar Omega microplate reader (BMG Labtech, Ortenberg, Germany). The decline in absorbance per sample was recorded and percentage quenching of DPPH• was calculated based on the observed decrease in absorbance using the formula: % inhibition = $\left[\left(A_{0}-A_{1}\right)÷A_{0}\right]\times 100\text{,}$ with A_0_ the absorbance value of the DPPH• blank and A_1_ the absorbance value of the sample.

#### Determination of total phenolics

Total phenolic content was measured according to Ainsworth and Gillespie (2007). After grinding, 20 mg frozen plant powder were added to 2 ml of ice-cold 95% methanol (v:v). Samples were incubated at room temperature for 48 h in the dark and then centrifuged at 13,000×*g* for 5 min. 100 μl of the supernatant were mixed with 200 μl 10% Folin and Ciocalteu’s phenol reagent and 800 ml 700 mM Na_2_CO_3_. After incubation at room temperature for 2 h, the absorbance was measured spectrophotometrically at 765 nm using a FLUOstar Omega microplate reader (BMG Labtech, Ortenberg, Germany). A standard curve from 0 to 1 mM gallic acid from Sigma-Aldrich (St. Louis, United States) was conducted to determine total phenolics as gallic acid equivalents.

#### Determination of protein content

The total soluble protein content in the leaves was determined by the Bradford (1976) method. For this, 100 mg ground fresh leaf material were extracted with 1.5 ml of extraction buffer containing 25 mM HEPES (pH 7.8) and 0.1 mM EDTA. After centrifugation at 15,000×*g* for 15 min, 0.05 ml of the supernatant was added to 2.5 ml Bradford Reagent (Sigma-Aldrich, St. Louis, United States). After 5 min of reaction time, the absorbance was measured spectrophotometrically at 595 nm using a FLUOstar Omega microplate reader (BMG Labtech, Ortenberg, Germany). To determine the protein content, the values were calculated from a standard curve of 0 to 2 μg μl^−1^ Bovine Serum Albumin (Merck KGaA, Darmstadt, Germany).

#### Determination of salicylic acid by UHPLC–MS analysis

One gram of frozen leaf material per sample was ground with liquid nitrogen. For extraction, 5 ml 80% (v:v) methanol was added. The samples were treated with a Miccra D-9 homogenizer (MICCRA GmbH, Heitersheim, Germany) for 1 min and then placed into a Sonorex RK510 ultrasonic bath (BANDELIN electronic GmbH & Co. KG, Berlin, Germany) for 15 s. Subsequently, 2 ml of the suspension were centrifuged at 10,000×*g* for 5 min. 350 μl of the supernatant were added to 700 ml ultrapure water, mixed, and centrifuged again at 12,000 rpm for 5 min. The supernatant was then filtered with a Chromafil disposable filter O-20/15 MS (MACHEREY-NAGEL GmbH & Co. KG, Düren, Germany). Analytical UHPLC–MS analysis was performed on a Velos LTQ System (Thermo Fisher Scientific, Waltham, United States). Separation was achieved on a Synergi Polar 4 μm 3 × 150 mm column. The mobile phase consisted of 5% acetonitrile and 0.05% formic acid (solvent A), and acetonitrile LCMS grade (solvent B). The gradient used was: 95% A/5% B, 0–1 min; 10% A/90% B, 11–13 min; 95% A/5% B, 13.1 min; 95% A/5% B, 16 min. The flow rate was 0.5 ml min^−1^, and the injection volume was 1 μl. The SA standard was purchased from Merck KGaA (Darmstadt, Germany). The standard was prepared in concentrations of 0.1, 0.5, 1.0 mg L^−1^ with water/acetonitrile (70:30 v:v). Phytohormone content in μg g^−1^ was calculated using the formula:


$$\mathrm{Phytohormone content}[\mu {\mathrm{g g}}^{-1}]=\frac{\left[x\frac{\mathrm{\mu g}}{\mathrm{mL}}\times \left(\frac{1050\;\mathrm{\mu L}}{350\;\mathrm{\mu L}}\right)\times 5\mathrm{mL}\right]}{\mathrm{initial}\;\mathrm{weight}\;\left(1g\right)}.$$

### Experiments with *Gaeumannomyces graminis* f. sp. *tritici*

#### Experimental setup

The greenhouse experiment with *Ggt* was conducted with the same winter wheat variety and the same temperature, lighting, and humidity settings as described in the previous experiment. Here, a fully randomized design with three replicates for each treatment was chosen (Supplementary Figure 1B). The soil used was a clayey loam derived from an Ap horizon of a long-term organically cultivated field site at the Hohenheim University agricultural research station Kleinhohenheim, Germany. Soil properties are listed in Supplementary Table 2. The soil was mixed with 30% (w:w) quartz sand to improve soil texture and facilitate root collection. After sieving the soil through a 5 mm mesh, it was fertilized with 80 mg kg^−1^ Ca(H_2_PO_4_)_2_, 150 mg kg^−1^ K_2_SO_4_, 50 mg kg^−1^ MgSO_4,_ and 200 mg kg^−1^ N of the three respective nitrogen forms calcium nitrate, ammonium sulfate, and calcium cyanamide. Additionally, non-nitrogen-fertilized soil was used as a control. The soil was then inoculated with *Ggt,* and 2 kg of soil were filled in each pot. The pots were inserted into a cooling system designed to regulate the root zone temperature of plants. The root zone temperature was set to 12°C to provide optimal conditions for *Ggt* infection.

The filled pots were kept moist at 70% WHC without plants for 2 weeks. Before planting, seeds were surface-sterilized by rinsing them in 10% H_2_O_2_ solution for 20 min. They were then stored overnight in an aerated 10 mM CaSO_4_ solution at 25°C in the dark. The next day, the seeds were placed on filter paper soaked with 4 mM CaSO_4_ solution. After 3 days of germination, the seedlings were transferred to the soil-sand substrate. Plants were cultivated for 4 weeks at 70% WHC and irrigated every other day. The day before harvest, irrigation of the plants was stopped to facilitate the separation of rhizosphere soil (RS) from bulk soil. Roots with adhering soil were gently separated from the bulk soil by hand agitation or mechanical shaking (Gouzou et al., 1993). The RS was then collected by mechanical shaking and careful separation of sticky soil from the roots. The soil samples were immediately frozen in liquid nitrogen and stored at −80°C. The pH of the RS was measured in 0.01 M CaCl_2_. After visual scoring of the roots, plant parts were dried separately at 40°C for 1 week and then weighted.

#### Inoculum production, inoculation, and disease assessment

The fungal strain *Gaeumannomyces graminis* var. *tritici* (DSM 12044) from the Leibniz Institute DSMZ (Braunschweig, Germany) was cultivated on potato dextrose agar plates for 18 days at 25°C until the plates were completely covered with the fungus. The soil was inoculated with 2 cm agar disks (10 per kg soil) by mixing them homogeneously into the soil-sand substrate. After harvesting, the roots were examined under a stereoscopic microscope (25× magnification) for intense vascular discoloration, a major characteristic of *Ggt*. The percentage of *Ggt* infected roots was estimated by counting the wheat plants’ discolored and non-discolored primary roots.

#### DNA extraction, amplicon library preparation, and sequencing

DNA extraction from the collected rhizosphere samples was performed using the DNeasy PowerLyzer PowerSoil Kit (Qiagen). The universal primer pair 27F/536R, which targets the V1-V3 region of the bacterial 16S rRNA gene was used to characterize the entire bacterial community (Weisburg et al., 1991; Leser et al., 2002). The primer pair ITS3/ITS4 was used to amplify the fungal internal transcribed spacer (ITS) rRNA region (White et al., 1990). PCR amplifications were performed in triplicate with a total volume of 50 μl reaction mix containing 1 μl of soil DNA template, 25 μl GoTaq G2 Master Mix (Promega, Mannheim, Germany), and 1 μl of each primer (25 μM). Amplicons were sequenced on an Illumina MiSeq instrument with 2 × 300 base pair kits by Eurofins Genomics Europe Sequencing (Constanz, Germany). Demultiplexing was performed with Illumina bcl2fastq 2.17.1.14 software following clipping of barcode and sequencing adapters. Reads with quality score of <20 were removed. DADA2 pipeline was used to determine amplicon sequence variants (ASV) from the raw sequences (Callahan et al., 2016). For bacterial sequences, forward and reversed reads were truncated at 270 and 140 bp, respectively. For fungal reads, forward and reversed reads were truncated at 260 and 160 bp, respectively. Alpha diversity metrics were calculated from the normalized sequence library, which was rarefied to 20,000 reads per sample for both microbial groups (bacterial and fungal samples). Rarefaction curve for both microbial groups reached plateau, indicated the sequencing depth was adequate (Supplementary Figure 2). Only ASVs that were detected in more than two samples were included in the data analyses. Bacterial 16S ASV representative sequences were classified using the naive Bayesian classifier (Wang et al., 2007) for Silva 138. Representative sequences for fungal ITS ASVs were classified using the naive Bayesian classifier (Wang et al., 2007) against Unite 8.3 reference database (Nilsson et al., 2019). All sequences were submitted to the European Nucleotide Archive (study accession numbers PRJEB52826 for bacterial and PRJEB52827 for fungal data).

### Statistics

Statistical analyses were performed using R version 4.1.1 (2021-08-10). The comparison of means was statistically analyzed with a two-factorial ANOVA followed by a Tukey’s HSD *post hoc* test (*p* < 0.05 significance level). Unless otherwise stated, data are presented as means ± SE. Normal distributions and variance homogeneities were checked by the Shapiro–Wilk test and by Levene’s test, as well as graphically against the predicted values by QQ-plots, histograms, and graphs of the residuals. If the normal distribution was not given, the data were transformed by taking the inverse of it. A “letter display” was used to visualize statistically significant differences in the means.

Differences in bacterial and fungal ASVs richness were compared using ANOVA followed by a Tukey’s HSD *post hoc* test. To assess differences in the bacterial and fungal microbiota structure between the different fertilization treatments, we first calculated Bray–Curtis dissimilarities using square-root transformed relative abundances (Hellinger transformation; Legendre and Gallagher, 2001). Permutational multivariate analysis of variances (PERMANOVA) based on the Bray–Curtis dissimilarity index was performed to analyze the effect of different N forms applications on the bacterial and fungal community structure, using 999 permutations for each test. Principal coordinates analysis (PCoA) based on the Bray–Curtis dissimilarity index was used to visualize the distribution of microbial community patterns in relation to the fertilization treatments. Linear discriminant analysis effect size (LEfSe) was applied to identify biomarker taxa explaining differences between the microbiota in the different N fertilization treatments (Segata et al., 2011).

## Results

### Leaf infection with *Blumeria graminis* f. sp. *tritici* and disease symptoms of powdery mildew

#### Plant performance with different N-fertilizers and *Bgt* infection

**Figure 1:**
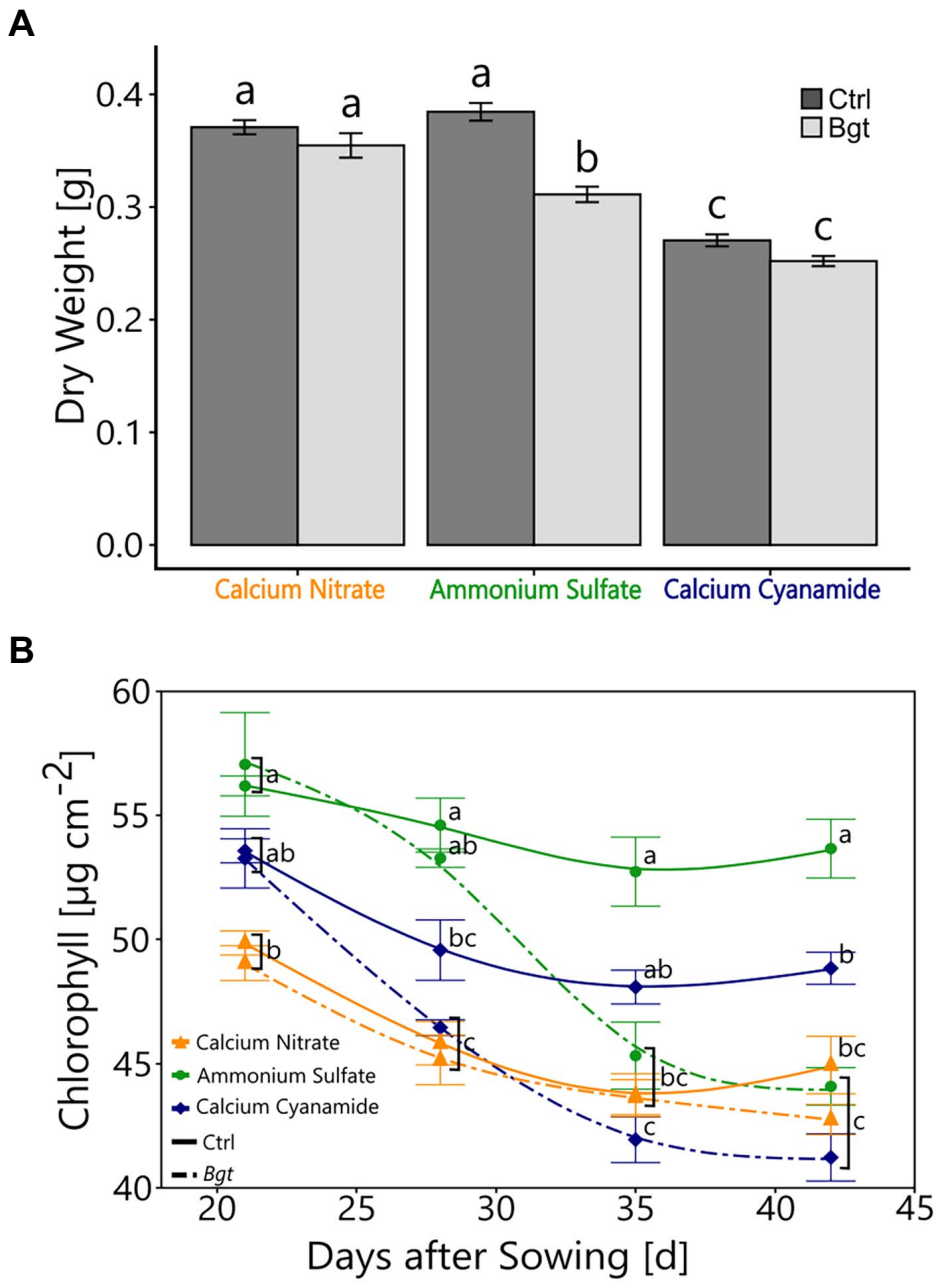
Plant performance with different N fertilizers and *Bgt* infection. **(A)** Plant dry weight (g) of winter wheat plants 28 days post-infection. **(B)** Chlorophyll content (μg cm^−2^) of winter wheat plants 21, 28, 35, and 42 days after sowing, treated with different nitrogen forms (calcium nitrate, ammonium sulfate, calcium cyanamide) under control conditions and *Bgt* infection. Data represent mean values of three biological replicates ± SE. The different lowercase letters indicate significant differences at 0.05 probability level according to Tukey multiple comparison test.

To determine whether fertilization with a specific N form and *Bgt* infection affected the plant performance, the dry weight and chlorophyll concentration of the plants were examined. *Bgt* infection led to a reduction in dry weight in ammonium-fertilized plants, but not in nitrate-and cyanamide-fertilized plants. Overall, cyanamide-fertilized plants showed a lower plant dry weight than nitrate- and ammonium-fertilized plants (Figure 1A). After about 2 weeks of *Bgt* infestation, the chlorophyll concentration of the plants in the ammonium- and cyanamide-fertilized plants started to decrease gradually after *Bgt* infection. The chlorophyll concentration of plants fertilized with nitrate was lower than with the other fertilizers, but remained unaffected by *Bgt* inoculation throughout the study period (Figure 1B).

#### Leaf disease symptoms by *Bgt* infection depend on nitrogen form

**Figure 2:**
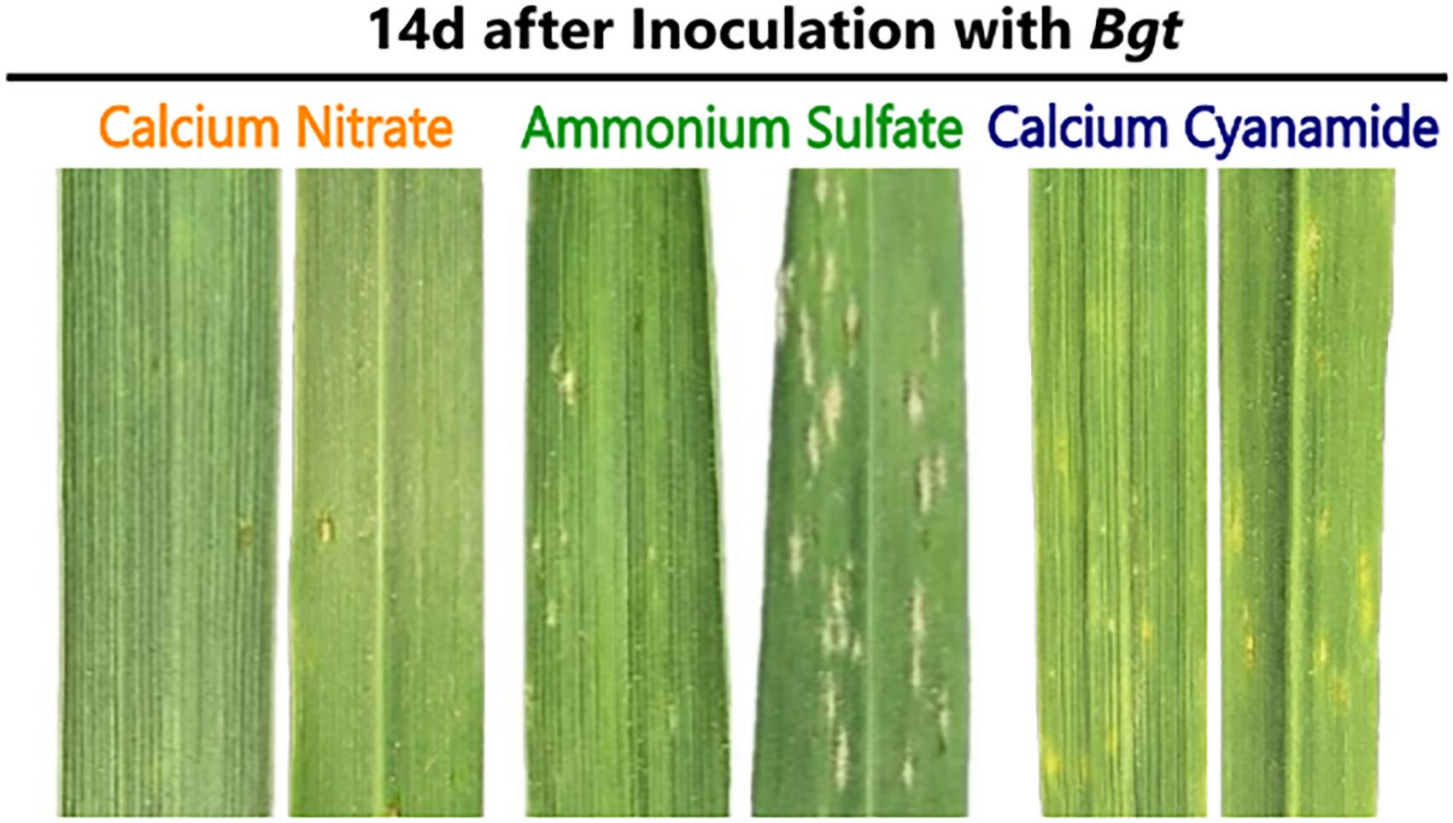
Powdery mildew infestation on the upper (left) and lower (right) surface of winter wheat leaves when fertilized with calcium nitrate, ammonium sulfate, or calcium cyanamide, 14 days (d) after *Bgt* infection.

*Bgt* infestation of wheat plants fertilized with different N forms was evaluated by visual assessment of the leaves. In this context, the disease index was calculated and, together with leaf images, the disease outbreak was documented. Tremendous differences were found between the fertilizer treatments 14 days post-infection (dpi). While nitrate-fertilized wheat plants showed hardly any visual disease symptoms, ammonium-fertilized leaves were already heavily infected. Microscopic analysis showed the presence of mycelium and conidial chains on these leaves. In addition, visual inspection indicated that cyanamide-fertilized leaves were less infected than ammonium-fertilized leaves (Figure 2). The disease index varied in all three fertilizer treatments 14 days after inoculation and gradually increased in ammonium- and nitrate-fertilized plants. Ammonium-fertilized leaves were nearly entirely covered with mycelium at 28 dpi, while nitrate-fertilized leaves were, by contrast, at a very low infection level. Ammonium fertilization increased the disease severity by 80% compared to nitrate fertilization and by 77.5% compared to cyanamide fertilization at 28 dpi. In cyanamide-grown plants, the disease index barely increased over time and at 21 and 28 dpi, and no differences to nitrate-fertilized plants were observed (Table 1).

**Table 1:** Disease index of powdery mildew on winter wheat cultivar Emerick treated with different nitrogen forms (calcium nitrate, ammonium sulfate, calcium cyanamide) at 14, 21, 28 days post-infection (dpi).

Nitrogen fertilization	Powdery mildew (*Bgt*)		
	Disease index		
	14 dpi	21 dpi	28 dpi
Calcium nitrate	0a	4.44a	17.78a
Ammonium sulfate	44.44c	60b	88.89b
Calcium cyanamide	15.56b	15.56a	20a

Data represent mean values of three biological replicates ± SE. The different lowercase letters indicate a significant difference at 0.05 probability level according to Tukey multiple comparison test.

#### Analysis of mineral nutrients

Wheat leaves were analyzed for important macro-and micronutrients after 28 dpi to determine the nutritional status of the plants under control conditions and *Bgt* infection. Nitrate-fertilized plants had slightly lower nitrogen concentrations in comparison with cyanamide-fertilized plants, but nitrogen status was in all treatments above the critical value of 4.4% N (Justes et al., 1994), indicating no N deficiency. Regardless of *Bgt* infection, higher calcium concentrations were encountered in nitrate-fertilized plants compared to ammonium- and cyanamide-fertilized plants. Plants fertilized with ammonium had the highest P concentrations compared to the other fertilizations under non-infected conditions. With *Bgt* infection, nitrate-fertilized plants had higher magnesium and manganese concentrations, although all nutrients were at sufficiency levels (Bergmann, 1983). The fertilization regime and *Bgt* infection had no effect on the concentrations of carbon, sulfur, potassium, iron, and zinc (Supplementary Table 3).

#### Defense-related compounds with different N-fertilizer and *Bgt* infection

**Figure 3:**
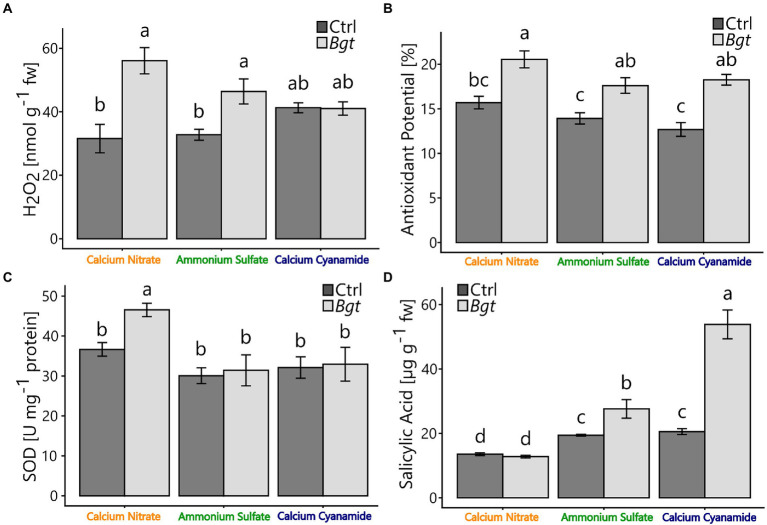
**(A)** Hydrogen peroxide (H_2_O_2_) (nmol g^−1^ fw), **(B)** antioxidant potential (%), **(C)** superoxide dismutase (U mg^−1^ protein), and **(D)** endogenous concentrations of salicylic acid (μg g^−1^ fw) of winter wheat plants after 28 dpi treated with different nitrogen forms (calcium nitrate, ammonium sulfate, calcium cyanamide) under control conditions (black bars) and *Bgt* infection (gray bars). Data represent mean values of three biological replicates ± SE. The different lowercase letters indicate significant differences at 0.05 probability level according to Tukey multiple comparison test.

To gain insight into plant defense during pathogen infection, key defense-related compounds, including hydrogen peroxide (H_2_O_2_) concentration, antioxidant potential, superoxide dismutase (SOD) activity, and salicylic acid concentration, were determined. Here, the leaf hydrogen peroxide concentration increased by 77% (nitrate) and 29% (ammonium) compared to the non-infected controls. However, no differences were measured in calcium cyanamide-fertilized plants with or without *Bgt* infection. Nevertheless, calcium cyanamide-fertilized plants tended to have higher leaf hydrogen peroxide concentrations under control conditions (Figure 3A). SOD activity was higher in *Bgt* infected, nitrate-fertilized plants, while ammonium-and cyanamide-fertilized plants infected with *Bgt* showed no differences in SOD activity compared to their respective non-infected controls (Figure 3C). For all fertilizer treatments, the antioxidant potential was consistently higher in *Bgt* infected plants (Figure 3B). Interestingly, plants infected with *Bgt* showed 42% and 162% higher salicylic acid concentrations with ammonium and cyanamide. In contrast, salicylic acid was not increased in nitrate-fertilized *Bgt* infected plants and was lower overall compared to the other fertilizer treatments (Figure 3D). Total protein and total phenolics were independent of fertilizer and *Bgt* infection (Supplementary Figure 3).

### Experiments with *Gaeumannomyces graminis f.* sp. *tritici (Ggt)*

#### Plant performance and root disease infestation by *Ggt*

**Figure 4:**
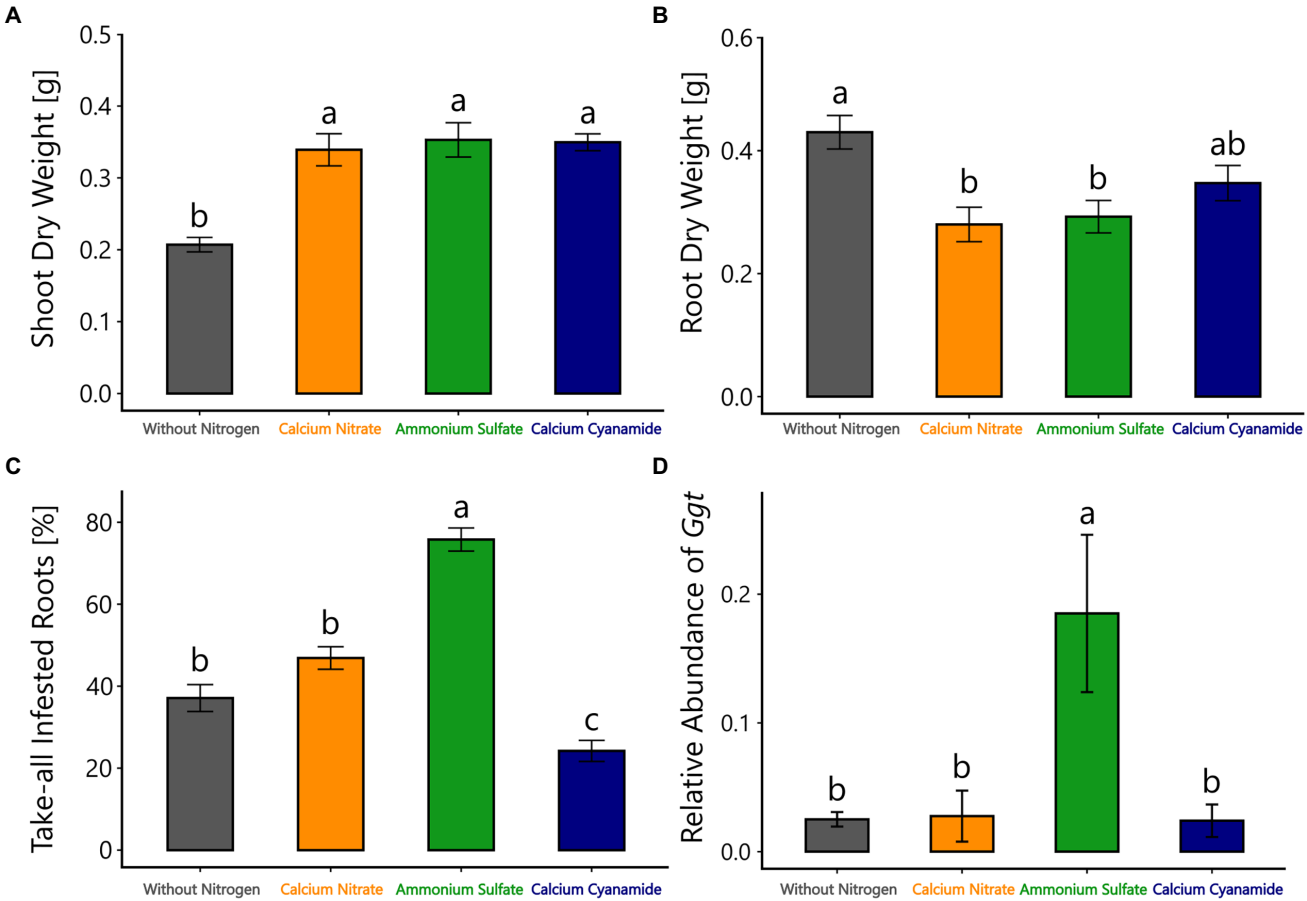
**(A)** Shoot and **(B)** root dry weight (g), **(C)** take-all infested roots (%), and **(D)** relative abundance of *Ggt* in the rhizosphere of winter wheat from the amplicon sequencing data, 28 days after sowing, treated with different nitrogen forms (without nitrogen, black bar; calcium nitrate, orange bar; ammonium sulfate, green bar; calcium cyanamide, blue bar) under *Ggt* infection. Data represent mean values of three biological replicates ± SE. The different lowercase letters indicate significant differences at 0.05 probability level according to Tukey multiple comparison test.

The performance of *Ggt*-infested plants fertilized with different N forms as well as without N was determined by measuring root and shoot dry weight. In line with our expectations, wheat plants without additional N fertilizer had lower shoot dry weight in comparison with the ones fertilized with different N forms at 28 days after sowing, but shoot dry weight was similar with all different N forms (Figure 4A). By contrast, the root dry weight was higher when plants were not N-fertilized, compared to nitrate-or ammonium-fertilized plants (Figure 4B).

*Ggt* infestation was determined by visual assessment of take-all infested roots and determination of the relative abundance of *Ggt* in the wheat rhizosphere. Plants fertilized with ammonium were found to have a high infestation of take-all, indicated by a 75% discoloration of the primary roots. A lower infestation of 46% and 37% of *Ggt* infected roots was observed in nitrate-fertilized plants and plants without N fertilization, respectively. Cyanamide-fertilized plants were the least infected, with 24% of the primary roots being discolored (Figure 4C). In addition, a higher relative abundance of *Ggt* in the rhizosphere was observed in the ammonium treatment as shown by the amplicon sequencing data. In other treatments, the relative abundance of *Ggt* in the rhizosphere was similarly low (Figure 4D).

The pH of the rhizosphere was 6.73 without N fertilization, 6.81 with nitrate and 6.13 with ammonium, while cyanamide raised pH to 7.12 (Supplementary Table 4).

#### Microbial composition of the wheat rhizosphere

Metabarcoding was used to obtain information on bacterial and fungal communities and their composition in the rhizosphere of wheat. A total of 606,069 16S bacterial and 557,603 ITS fungal high-quality reads were recovered from the 12 rhizosphere samples, which clustered into 1,914 bacterial and 518 fungal amplicon sequence variants (ASVs). Overall, bacterial sequences were affiliated with 22 phyla (Supplementary Figure 4), 61 classes, 131 orders, 188 families, and 301 genera. Actinobacteriota were the most abundant phylum, comprising ~36.7% of the total bacterial reads (603 ASVs), followed by Proteobacteria (24.7% of reads; 419 ASVs) and Chloroflexi (10.2%, of reads; 237 ASVs). The most abundant bacterial genera detected across all soil samples were *Nocardioides* (3.8%), KD4-96 (3.4%), *Bradyrhizobium* (2.4%), *Gemmatimonas* (2.2%) and *Flavobacterium* (2.2%). The fungal sequences were associated with 8 phyla (Supplementary Figure 5), 28 classes, 70 orders, 127 families, and 130 genera. Ascomycota (92.7% of reads; 382 ASVs) was the dominant fungal phylum, followed by Basidiomycota (3.7% of reads; 55 ASVs), and Mucoromycota (1.9% of reads; 15 ASVs). Each of the other fungal phyla represented <1%. *Fusarium*, *Blumeria,* and *Cladosporium* were the most abundant genera identified in this study, accounting for 11.8%, 5.7%, and 4.3%, of the total fungal reads, respectively.

#### Effect of N fertilizer form on the bacterial and fungal alpha and beta diversity

**Figure 5:**
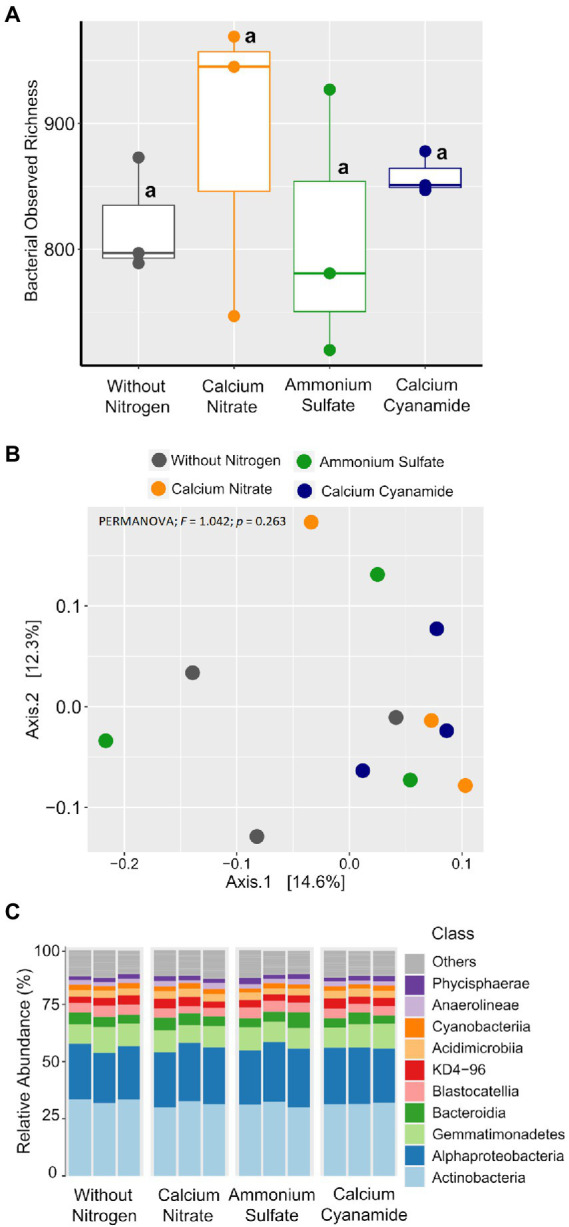
Box plots of the bacterial observed richness. **(A)** Principal coordinates analysis (PCoA) based on Bray–Curtis dissimilarity. **(B)** Colors: without nitrogen, black; calcium nitrate, orange; ammonium sulfate, green; calcium cyanamide, blue. Effects of N fertilization were tested with PERMANOVA (based on Hellinger transformed data and the Bray–Curtis distance measure). **(C)** Mean relative abundance of the main classes of the bacterial communities detected. The same lowercase letters indicate no significant differences at 0.05 probability level according to Tukey multiple comparison test.

**Figure 6:**
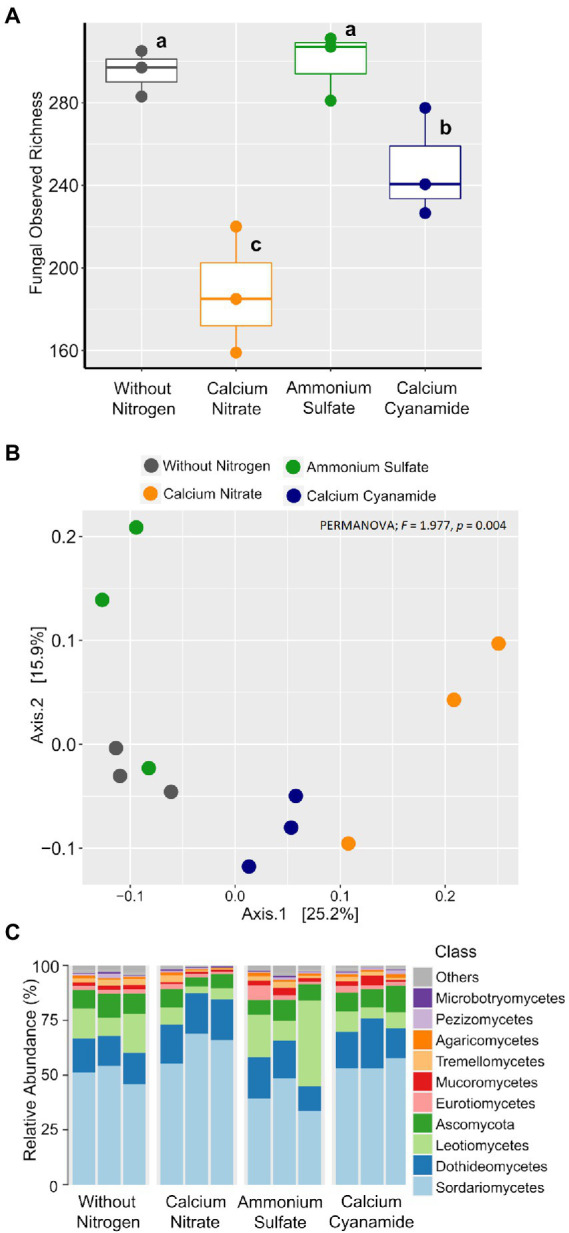
Box plots of the fungal observed richness. **(A)** Principal coordinates analysis (PCoA) based on Bray–Curtis dissimilarity. **(B)** Colors: without nitrogen, black; calcium nitrate, orange; ammonium sulfate, green; calcium cyanamide, blue. Effects of N fertilization were tested with PERMANOVA (based on Hellinger transformed data and the Bray–Curtis distance measure). **(C)** Mean relative abundance of the main classes of the fungal communities detected. The different lowercase letters indicate significant differences at 0.05 probability level according to Tukey multiple comparison test.

To investigate the effect of N fertilization on the bacterial and fungal communities associated with the wheat rhizosphere, we first calculated the observed richness and Bray–Curtis dissimilarity as proxies for alpha and beta diversity metrics, respectively, for both microbial groups. Bacterial richness ranged from 720 to 969 ASVs and it did not differ among the four fertilization N treatments (Figure 5A). Likewise, bacterial community structure was not affected by N application and N form (PERMANOVA; *F* = 1.042; *p* = 0.263). At a higher taxonomic level, no differences in the proportion of the main bacterial classes were observed among N treatments (Figures 5B,C). Fungal richness ranged from 161 to 312 ASVs and it was higher in soil without any fertilization and in the ammonium treatment, while the soil amended with nitrate showed the lowest value in richness (Figure 6A). Fungal community structure was affected by N fertilizer (PERMANOVA; *F* = 1.977, *p* = 0.004), which accounted for 42.6% of community variation. Principal coordinate analysis (PCoA) confirmed the notable effect of differential N forms on fungal community structure, displaying a clear separation of the different N treatments along with both coordinates, which together explained more than 40% of the variation (Figure 6B).

#### Compositional shifts in fungal, but not bacterial, community assembly by N fertilizer

To better understand how microbial community assembly was affected by the application of the different N forms, we sought to explore the composition and structure of the wheat rhizosphere microbiota. Looking at the bacterial community associated with the four N treatments, we observed that they were dominated by the same bacterial groups across different taxonomic ranks (Figure 5C). Indeed, the unique ASVs detected in each N treatment accounted for a marginal proportion of sequences (Supplementary Figure 6), while more than 80% of the total bacterial sequences were found in all treatments (Supplementary Figure 7). Many of these shared ASVs were affiliated with several representative families and genera (>1% abundance), most of which did not show abundance shifts across N treatments (Supplementary Figure 7). Furthermore, only a handful of low representative taxa were found as indicative biomarkers of the four fertilization treatments (Supplementary Figure 8). Overall, these findings indicated high similarities in bacterial community composition and structure among N treatments, corroborating the results obtained by beta diversity analysis.

**Figure 7:**
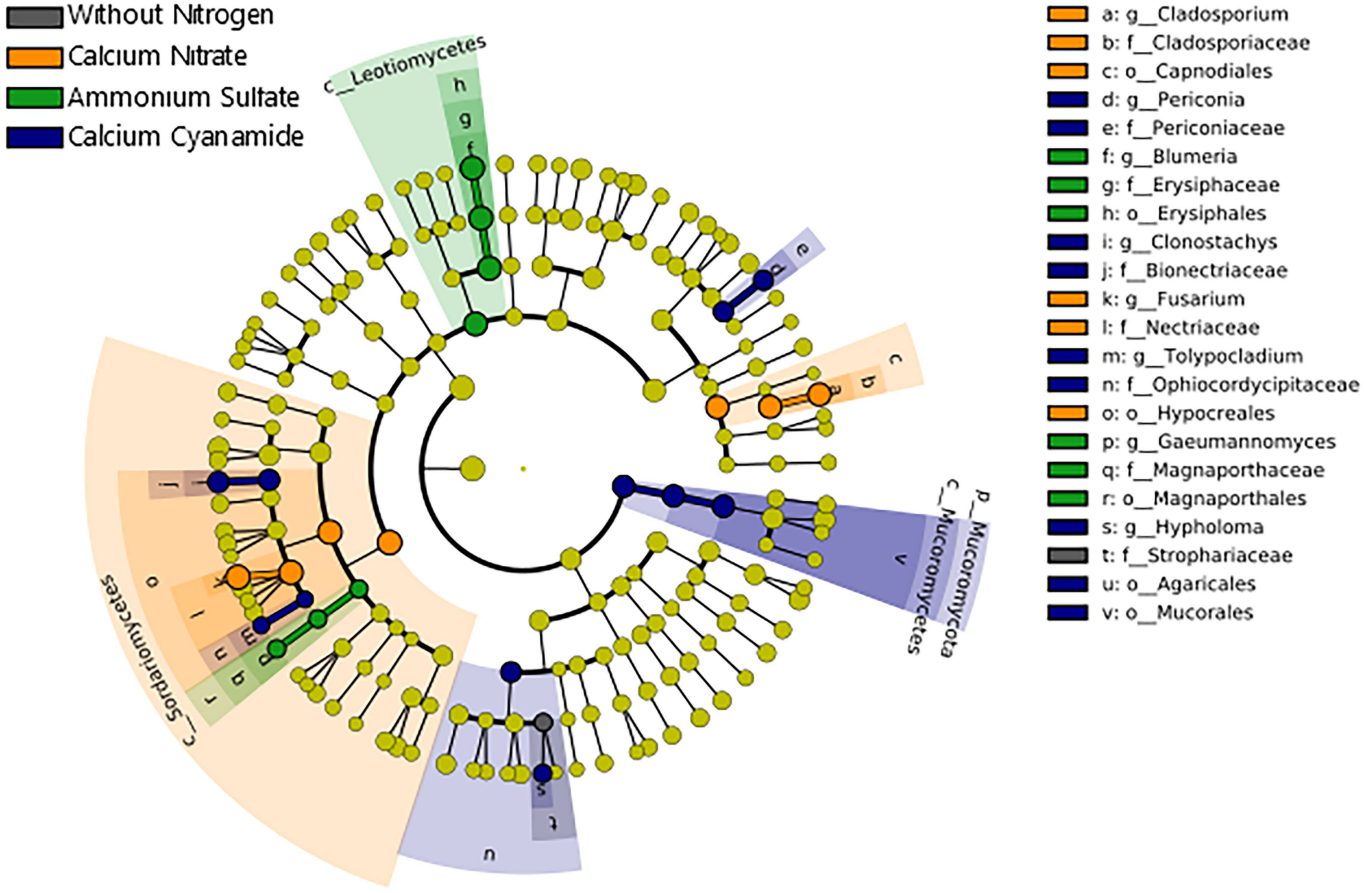
Cladogram illustrating the taxonomic groups explaining the most variation among the fungal taxa. Each ring represents a taxonomic level, with phylum (p_), class (c_), order (o_), family (f_), and genus (g_) emanating from the center to the periphery. Each circle is a taxonomic unit found in the dataset, with circles or nodes shown in colors (other than yellow) indicating where a taxon was significantly more abundant. Black: without nitrogen, orange: calcium nitrate, green: ammonium sulfate, blue: calcium cyanamide.

Contrarily to the bacteria, the differences observed in fungal beta diversity between the four N treatments were reflected by substantial shifts in the abundance of several fungal taxonomic groups (Figure 6C; Supplementary Figure 9). Linear discriminant analysis (LDA) effect size (LEfSe) further confirmed these observations, identifying 26 fungal biomarker taxa associated with the different N forms applied (Figure 7; Supplementary Figure 10). For instance, enrichments within the Ascomycota groups Sordariomycetes (class) and Capnodiales (order) characterized the nitrate-fertilized samples. On the other hand, cyanamide fertilization increased the abundance of taxa affiliated with the Mucoromycota phylum and the Agaricales order (Basidiomycota). The fungal community identified in ammonium-fertilized soil was represented by a higher proportion of members within the Ascomycota class Leotiomycetes and Magnaporthales order. At a finer taxonomic ranking, we observed increased abundance of fungal groups associated with the Ascomycota genera *Fusarium* and *Cladosporium* in nitrate-fertilized soil. Conversely, the Ascomycota genera *Clonostachys*, *Periconia,* and *Tolypocladium*, together with the Basidiomycota genus *Hypholoma* were found as biomarkers for the cyanamide treatment. Intriguingly, we found that ammonium fertilization promoted the build-up of fungal pathogens affiliated to the genera *Blumeria* and *Gaeumannomyces*, as they were more abundant in this fertilization treatment compared to the others. Notably, the mean abundance of the genus *Gaeumannomyces* was approximately six times higher in the ammonium treatment than in the other N fertilized soils (Figure 4D), reflecting the higher root infection rate observed in this treatment (Figure 4C). Finally, fungal taxa associated with the Basidiomycota family Strophariaceae were found as a biomarker for the treatment without N fertilization.

## Discussion

In this study, we investigated the interaction between N forms (nitrate, ammonium, and cyanamide) and two economically important specialist fungal pathogens: an obligate biotroph, *B. graminis* f. sp. *tritici* and a soil-borne necrotroph, *Gaeumannomyces graminis* f. sp. *tritici*. We conducted greenhouse experiments in which wheat was deliberately inoculated with these pathogens. This allowed us to examine winter wheat plants’ growth and key metabolite defense reactions during pathogen interaction. Our findings highlighted that wheat leaves inoculated with the foliar pathogen *Bgt* were comparatively less infested when fertilized with nitrate or cyanamide than with ammonium. Likewise, soil inoculation with the fungal pathogen *Ggt* revealed a higher percentage of infected roots in ammonium-fertilized plants. Additionally, metabarcoding data revealed that bacterial communities were merely affected by N fertilization, whereas the fungal community assemblage was differently shaped by N fertilization type. Specifically, we observed a dramatic increase in the abundance of fungal pathogens associated with cereal crop diseases along with ammonium fertilization. Overall, our study demonstrates that particular N fertilization treatments could select, promote or reduce specific groups of beneficial or detrimental soil microorganisms, and give indications that ammonium fertilization facilitates the build-up of plant pathogens in winter wheat.

### Ammonium fertilization increased wheat’s susceptibility to *Bgt*, while nitrate and cyanamide fertilization could reduce the disease infestation

N fertilization in the form of ammonium made the plant most susceptible to *Bgt* infection (Table 1; Figure 2). Besides the visual scoring of the plant’s disease severity, also leaf chlorophyll concentration, a common indicator of healthiness and plant-pathogen interactions (Rolfe and Scholes, 2010; Li et al., 2017; Yang and Luo, 2021), decreased after 3 weeks of infection (Figure 1B). Additionally, the *Bgt* infection resulted in lower dry weight (Figure 1A), confirming that ammonium-fertilized plants suffered severe pathogen infestation. The higher susceptibility of ammonium-fertilized shoots could result from the overall highest chlorophyll concentrations measured in ammonium-fertilized leaves in early infection stages. The resulting leaf metabolism might be more favorable for leaf pathogens. Defense-related metabolites or enzymatic activities were affected in ammonium-fertilized plants infected with *Bgt* and showed an increase in H_2_O_2_, antioxidant potential, and SA (Figure 3). Only SOD activity was not enhanced in infected plants. Overall, ammonium-fertilized plants apparently defended themselves against the pathogen attack, but this did not alter their susceptibility. In the literature, ammonium fertilization is often associated with increased susceptibility to various pathogens (Bolton and Thomma, 2008; Mur et al., 2017). This was attributed to reduced NO levels, as nitrate reductase (NR) is inactive without nitrate (Planchet et al., 2005). *Bgt* spread on the leaf surface could also be enhanced by higher P concentrations that we measured in ammonium-fertilized plants (Supplementary Table 3). Leaf pathogens, like *Bgt*, are known to take advantage of nutrients released into the apoplast of epidermal cell walls (Mustafa et al., 2016). The higher leaf P concentrations can be attributed to decreased rhizosphere/soil pH, resulting from nitrification and the intentional release of protons in exchange for ammonium by the root (Grunes et al., 1958; Riley and Barber, 1971).

The fertilization of nitrate resulted in the lowest *Bgt* infestation compared to the other fertilizer treatments (Figure 2). Besides the low visual scoring of the plant’s disease severity, chlorophyll concentration and dry weight (Figure 1A) did not decrease between control and inoculated plants (Figure 1B). Nevertheless, leaves of nitrate-fertilized plants generally had lower chlorophyll concentrations than leaves of plants fertilized with the other N fertilizers. This can be attributed to the slightly lower percentage of N measured in the leaves of nitrate-fertilized plants (Supplementary Table 1), which is highly correlated with the chlorophyll concentration (Bojović and Marković, 2009). A lower nitrogen concentration in the plant is frequently related to a higher tolerance against leaf pathogens and could be an explanation for the lower infestation rate (Huber and Watson, 1974; Sun et al., 2020). Furthermore, nitrate-fertilized shoots exhibited an enhanced defense response after contact with the pathogen, as evidenced by elevated H_2_O_2_ concentration, SOD activity, and antioxidant potential (Figure 3). Especially H_2_O_2_ is locally needed at the infection site to promote pathogen defense *via* the oxidative burst (Bolwell, 1999; Apel and Hirt, 2004; Shetty et al., 2007). H_2_O_2_ is required for polysaccharide-protein cross-linking and lignification, processes involved in fungal penetration defense (Shetty et al., 2007). Also, H_2_O_2_ is produced during the oxidative deamination of polyamines by a series of amine oxidases (Cona et al., 2006). Gupta et al. (2013) detected high levels of polyamides in nitrate-fertilized plants, which increased hypersensitive response-associated resistance in cereals against *B. graminis* (Cowley and Walters, 2002a,b). Additionally, various enzymatic and non-enzymatic detoxification mechanisms protect the surrounding cell areas from oxidative stress and are regulated by nitrogen nutrition (Sun et al., 2020). Though nitrate promotes the formation of protective substances in the plant in presence of the pathogen to resist the *Bgt* infestation, we observed low levels of SA in nitrate-fertilized control and infected plants (Figure 3D). This might partially be explained by the low level of *Bgt* infection with nitrate. Additionally, higher concentrations of the nutrients Mg, Mn, and Ca were found with nitrate (Supplementary Table 2), potentially due to charge balance requirements. While the role of Mg on plant diseases is mainly *via* indirect mechanisms, no direct effects on *Bgt* infection are known (Huber and Jones, 2013). In contrast, as a cofactor of the SOD, Mn participates in the plant’s defense against oxidative stress and additional fertilization of Mn can reduce the infection of *Bgt* in wheat (Colquhoun, 1940). Higher Ca concentrations can be partially explained by the fertilization of nitrate in form of calcium nitrate. Nevertheless, cyanamide was also applied as calcium cyanamide and showed lower concentrations of leaf *Ca.* Higher concentrations of Ca in the shoot tissue, especially in pectin fractions of the cell wall, may also help the plant to resist against pathogens. Ca^2+^ sensors and their target proteins are part of defense-signaling pathways that interact downstream with effectors that modulate numerous biochemical and cellular functions in pathogen defense responses (Zhang et al., 2014).

N fertilization in form of cyanamide decreases the *Bgt* infection in comparison with ammonium fertilization as well (Table 1). Under field conditions, Heitefuss and Brandes (1970) already reported a reduction in *Bgt* infection in wheat when fertilizing cyanamide in comparison with other mineral fertilizers, such as ammonium nitrate. Besides the enhanced *Bgt* resistance, our results showed that cyanamide fertilization reduced plant biomass and height under control conditions (Figure 1A). This might indicate that plants were stressed by the phytotoxicity of cyanamide and its detrimental effect on plant growth, which is usually stronger in dicots (Soltys et al., 2012; European Chemicals Agency, 2018). The stress indicator H_2_O_2_ might confirm these assumptions, as it was higher in the cyanamide-fertilized control in comparison with the other fertilizers and remained similar in plants infected with *Bgt* (Figure 3A; Saxena et al., 2016). The phytotoxic effect of cyanamide is often observed in young plants when further degradation of this compound is not yet sufficiently advanced (Temme, 1948). A common plant response to the increased oxidative stress promoted by cyanamide application is the increase in antioxidant plant potential (Soltys et al., 2012; Sabatino et al., 2019), but SOD activity was not higher. Interestingly, SA was strongly elevated in this N treatment infected with the pathogen (Figure 3D). Collectively, these findings suggested a prompted activation of the plant defense response and the establishment of systemic acquired resistance with cyanamide applications (Grant and Lamb, 2006; Bari and Jones, 2009).

Independent from nitrogen nutrition, Jawhar et al. (2017) found that cereals infected with *Blumeria graminis* showed higher levels of total SA in the leaves. After *Bgt* inoculation, Xin et al. (2012) reported an altered expression of genes encoding key enzymes in the phenylpropanoid pathway such as cytochrome P450 monooxygenase which converts benzoic acid to SA. This indicates that SA plays a positive role in signaling events during fungal infection and is involved in an early local defense response at the primary *Blumeria graminis* infection site. Additionally, SA plays a central role in systemic resistance to pathogen infection. In this signaling pathway, NO is also known to be an integral component, which is closely related to nitrate nutrition due to the catalysis of nitrate reductase (Mur et al., 2005, 2013; Chen et al., 2014).

### Ammonium fertilization promoted *Ggt* root infestation and the build-up of important soil borne pathogens compared to other N forms

In the second set of experiments that involved soil inoculation of the fungal pathogen *Ggt*, causing take-all disease in wheat, the different N fertilization treatments resulted in similar shoot and root dry weights, indicating that application of different N forms substantially did not influence plant biomass. However, the non-N fertilized control treatment led to higher root dry weight and reduced shoot dry weight (Figures 4A,B). Such change in root/shoot ratio is commonly observed in N-deficient plants, as they may attempt to increase N availability *via* investing in belowground biomass (Luo et al., 2020), a process that is disease-independent. Intriguingly, cyanamide fertilization produced the lowest *Ggt* infection rate, with no negative impact on plant performance. This is the opposite of what we observed in the first experiment with the shoot pathogen. Perhaps, cyanamide lost its possible phytotoxicity in these experiments, as the fertilized pots were incubated for 2 weeks prior to plant sowing and soil microorganisms need approximately this time window to convert cyanamide into urea, ammonium, and finally nitrate (Temme, 1948).

Notably, the highest take-all infestation was again observed in ammonium-fertilized plants (Figure 4C). *Ggt* abundance in the wheat rhizosphere was higher in this treatment compared to the other ones (Figure 4D). In contrast to our findings, it has been reported that nitrogen fertilization in form of ammonium can suppress root infections promoted by *Ggt*. This process was mainly attributed to the direct inhibition of ectotrophic hyphal growth of *Ggt* by a decrease in pH of wheat root surface by the application of ammonium fertilizer (Smiley and Cook, 1973; Smiley, 1978). The decrease in rhizosphere pH associated with ammonium could also increase the availability of micronutrients, such as Mn, which is known to influence host susceptibility to take-all, especially in cereal crops (Huber and Wilhelm, 1988; Huber, 1993; Ownley et al., 2003). However, such a suppressive pH effect was only visible after 7 weeks from the pathogen inoculation (almost twice our experiment running time), and it was removed by fumigating the soil before *Ggt* inoculation, implying that *Ggt* suppression is highly dependent on soil microbes. Indeed, the soil and plant-associated microbiota play a crucial role in plant growth, plant health, and stress tolerance, including pathogen control (Arif et al., 2020; Song et al., 2020). There is also evidence that wheat cultivars differ in their ability to support the suppression of take-all disease promoted by *Ggt* and other microbes, suggesting that this disease likely involves a complex interaction between the take-all fungus, wheat host genotype, and soil and plant-associated microbiota (Yang et al., 2018). In this context, several studies on *Ggt*-suppressive soils, soils that prevent *Ggt* establishment or reduce disease incidence (Jara and Elizondo, 2011) even in presence of a susceptible host plant, and favorable soil or climate conditions, have shown that native soil microorganism activity can be pivotal in *Ggt* disease suppression (Durán et al., 2017). Although the mechanisms of such soil suppressiveness are not fully understood, it has been often reported that *Ggt*-suppressive soils are characterized by a decrease in rhizosphere pH together with the enrichment of populations of root-associated *Pseudomonas* spp. (Weller et al., 2002). In particular, this may target *Pseudomonas fluorescens* strains, which can produce the antifungal polyketide 2,4-diacetylphlorogucinol (2,4-DAPG; Raaijmakers and Weller, 1998; Raaijmakers et al., 1999; Ownley et al., 2003). In our experiment, ammonium fertilization decreased rhizosphere pH (Supplementary Table 4), but *Ggt* disease was not suppressed, and *Pseudomonas* spp. were not increased in abundance and accounted only for a relatively small proportion of bacterial sequences (less 1% of total reads). In this line, our results showed that N fertilization merely affected bacterial community composition and structure, which agrees with insignificant changes found with a meta-analysis on agricultural systems (Geisseler and Scow, 2014) and several other studies where minimal effect was found of mineral fertilizers on soil bacteria community assembly in the very short term (few weeks from their applications; Marschner et al., 2001; Stark et al., 2007; She et al., 2018) or even long term (Liu and Ludewig, 2019). Moreover, a very recent work highlighted that short-term nitrogen fertilization had a greater impact on fungal community composition and structure than on bacterial communities in different compartments along the soil-root continuum (bulk soil, rhizosphere, and endophytic environment) of maize (Bai et al., 2022). Our results supported these observations, as we found that the fungal community was highly responsive to N fertilization, which partially validated our hypotheses. Indeed, differences in alpha and beta diversity were evident among the different N treatments, and distinct fungal assemblages characterized each N fertilized soil. In the short-term, fungal communities may respond to nitrogen fertilization as a disturbance, with consequent profound shifts in community assembly (Paungfoo-Lonhienne et al., 2015; Treseder et al., 2018; Cai et al., 2021; Castle et al., 2021). However, the type of N form employed might play a crucial role in selectively increasing or decreasing specific fungal groups with important ecological and agricultural consequences, such as favoring pathogenic over mutualistic fungal taxa, or vice versa (Huber and Watson, 1974; Francioli et al., 2016; Gu et al., 2020; Lekberg et al., 2021). Here, we observed a pronounced effect of ammonium fertilization in the build-up of important fungal pathogens such as *Ggt* and *Blumeria graminis* (Figure 7). Surprisingly, the relative abundance of *Ggt* was almost sixfold higher in the ammonium-fertilized wheat rhizosphere than in the other N amended soils, highlighting the detrimental effect of this N form on the wheat root-associated microbiota. The increase in plant susceptibility to pathogens from NH_4_^+^ fertilization has been ascribed to enhanced content of apoplastic sugar, amino acids, as well as γ-amino-n-butyric acid (GABA), which may represent efficient nutritional sources for the pathogen (Gupta et al., 2013; Mur et al., 2017). Furthermore, ammonium fertilization increased asparagine concentrations, which promote the development of *Rhizoctonia solani* in beet, and *Fusarium* wilt on tomato (Huber and Watson, 1974). However, more recent studies have illustrated that the interactions between mineral nitrogen and plant disease susceptibility are highly complex, and highlighted the role of endophytes, mainly fungal, in the reduction and suppression of take-all caused by *Ggt* (Maciá-Vicente et al., 2008; Liu et al., 2009; Durán et al., 2018; Gholami et al., 2019). Overall, our findings underlined the crucial role of N form in mineral fertilizers, as it can negatively influence the outcome of plant-pathogen interactions, as observed in this study with ammonium and winter wheat. To make a recommendation on which fertilizer strategy is preferable for winter wheat considering yield, disease control, safety and environmental impact, field trials need to be conducted at multiple locations.

## Data availability statement

The data presented in the study are deposited in the https://www.ebi.ac.uk/ena repository, accession numbers PRJEB52826 and PRJEB52827.

## Author contributions

NM and UL conceived and designed the experiments. NM and NP conducted the experiments and collected the data. NM, DF, MM, and GN analyzed the data. NM and DF wrote the manuscript with UL. All authors contributed to the article and approved the submitted version.

## Funding

This research was conducted within the projects “Agriculture 4.0 without Chemical-Synthetic Plant Protection” and the project DiControl within the program BonaRes (soil as a sustainable resource for the bio economy). Both projects are funded by the German Federal Ministry of Education and Research (BMBF), grant numbers 031B0731A and 031A560A-F, respectively.

## Conflict of interest

The authors declare that the research was conducted in the absence of any commercial or financial relationships that could be construed as a potential conflict of interest.

## Publisher’s note

All claims expressed in this article are solely those of the authors and do not necessarily represent those of their affiliated organizations, or those of the publisher, the editors and the reviewers. Any product that may be evaluated in this article, or claim that may be made by its manufacturer, is not guaranteed or endorsed by the publisher.
