# Supplementary material for: Ammonium fertilization increases the susceptibility to fungal leaf and root pathogens in winter wheat
Source: Front Plant Sci. 2022 Sep 9;13:946584. doi: 10.3389/fpls.2022.946584 (PMC9500508; doi:10.3389/fpls.2022.946584)
Supplement: Supplementary file 3 [file Data_Sheet_1.docx]

Ammonium fertilization increases the susceptibility to fungal leaf and root pathogens in winter wheat

Niels Julian Maywald^*^, Melissa Mang, Nathalie Pahls, Günter Neumann, Uwe Ludewig, Davide Francioli

Institute of Crop Science, Department of Nutritional Crop Physiology, University of Hohenheim, Stuttgart, Germany

*** Correspondence:**Niels Julian Maywald
niels.maywald@uni-hohenheim.de

**Supplementary Tables**

**Supplementary Table 1** | Chemical characterization of the topsoil used in the experiment with *Bgt*.

| pH^a^ |  | C_total_^b^ | C_org_^b^ | N^b^ |  | P^c^ | K^c^ | Mg^d^ |  | Fe^e^ | Mn^e^ | Zn^e^ |
| --- | --- | --- | --- | --- | --- | --- | --- | --- | --- | --- | --- | --- |
|  |  | % | | |  | mg 100 g^-1^ | | |  | mg kg^-1^ | | |
| 6.89 |  | 2.18 | 1.79 | 0.20 |  | 3.55 | 11.29 | 13.40 |  | 83.30 | 141.00 | 4.35 |

^a^pH = (CaCl_2_-suspension); ^b^C_total_, C_org_, N = (elementary analysis); ^c^P, K = (CAL-extract; VDLUFA); ^d^Mg = (CaCl_2_-extract; VDLUFA); ^e^Fe, Mn, Zn = (CAT-extract; VDLUFA)

**Supplementary Table 2** | Chemical characterization of the clayey loam used in the experiment with *Ggt*.

| pH^a^ |  | C_total_^b^ | C_org_^b^ | N^b^ |  | P^c^ | K^c^ | Mg^d^ |  | Fe^e^ | Mn^e^ | Zn^e^ |
| --- | --- | --- | --- | --- | --- | --- | --- | --- | --- | --- | --- | --- |
|  |  | % | | |  | mg 100 g^-1^ | | |  | mg kg^-1^ | | |
| 6.9 |  | 1.59 | 1.60 | 0.19 |  | 12 | 13 | 12 |  | 26.04 | 78.29 | 0.51 |

^a^pH = (CaCl_2_-suspension); ^b^C_total_, C_org_, N = (elementary analysis); ^c^P, K = (CAL-extract; VDLUFA); ^d^Mg = (CaCl_2_-extract; VDLUFA); ^e^Fe, Mn, Zn = (CAT-extract; VDLUFA)

**Supplementary Table 3 |** Nutrient analysis of winter wheat leaves after 28 dpi treated with different nitrogen applications (calcium nitrate, ammonium sulfate, calcium cyanamide) under control conditions and *Bgt* infection. Data represent mean values of three biological replicates ± SD. The different lowercase letters indicate a significant difference at 0.05 probability level according to Tukey multiple comparison test.

|  | **Ctrl** | | | ***Bgt*** | | |
| --- | --- | --- | --- | --- | --- | --- |
|  | **Calcium**  **Nitrate** | **Ammonium Sulfate** | **Calcium Cyanamide** | **Calcium**  **Nitrate** | **Ammonium Sulfate** | **Calcium Cyanamide** |
| **C [%]** | 40.83±0.46ab | 41.42±0.13a | 41.07±0.45ab | 40.36±0.34b | 41.40±0.08ac | 40.68±0.24bc |
| **N [%]** | 4.58±0.24a | 5.01±0.39ac | 5.67±0.13b | 4.91±0.06ad | 5.32±0.02bcd | 5.53±0.09bc |
| **S [%]** | 0.27±0.002a | 0.34±0.023ab | 0.36±0.005ab | 0.36±0.014ab | 0.38±0.020b | 0.39±0.008b |
| **P [mg plant^-1^]** | 0.42 ± 0.034bc | 0.65 ± 0.058a | 0.37 ± 0.012c | 0.41 ± 0.019bc | 0.50 ± 0.049b | 0.20 ± 0.010d |
| **Ca [mg plant^-1^]** | 2.39 ± 0.171a | 1.55 ± 0.082b | 0.87 ± 0.014c | 1.60 ± 0.153b | 1.19 ± 0.029d | 0.49 ± 0.032e |
| **K [mg plant^-1^]** | 12.12 ± 1.206ab | 13.34 ± 1.120a | 6.84 ± 0.471c | 10.77 ± 0.501bd | 9.69 ± 0.385d | 4.14 ± 0.108e |
| **Mg [mg plant^-1^]** | 0.41 ± 0.033ab | 0.46 ± 0.033a | 0.25 ± 0.008c | 0.46 ± 0.056a | 0.35 ± 0.015b | 0.14 ± 0.010d |
| **Fe [µg plant^-1^]** | 13.03 ± 1.584ab | 18.36 ± 1.307a | 8.73 ± 2.346b | 12.74 ± 1.390abc | 15.44 ± 5.935ab | 5.72 ± 1.442c |
| **Zn [µg plant^-1^]** | 5.52 ± 0.463ab | 8.50 ± 0.566a | 4.18 ± 0.026b | 5.74 ± 0.958ab | 6.07 ± 0.270a | 2.33 ± 0.170b |
| **Mn [µg plant^-1^]** | 8.51 ± 0.718a | 6.87 ± 1.378ab | 4.21 ± 0.346bc | 8.91 ± 0.926a | 6.35 ± 0.720ab | 2.37 ± 0.179c |

**Supplementary Table 4 |** Soil rhizosphere characterization of winter wheat plants, 28 days after sowing, treated with different nitrogen applications (without nitrogen, calcium nitrate, ammonium sulfate, calcium cyanamide) under *Ggt* infection. Data represent mean values of three biological replicates ± SD. The different lowercase letters indicate a significant difference at 0.05 probability level according to Tukey multiple comparison test.

|  | **Without Nitrogen** | **Calcium Nitrate** | **Ammonium Sulfate** | **Calcium Cyanamide** |
| --- | --- | --- | --- | --- |
| C [%] | 1.42 ± 0.02a | 1.47 ± 0.03a | 1.46 ± 0.07a | 1.45 ± 0.07a |
| N [%] | 0.16 ± 0.002b | 0.17± 0.004a | 0.18 ± 0.002a | 0.18 ± 0.005a |
| P [mg 100 g^-1^] | 11.96 ± 2.05a | 9.88 ± 1.01a | 8.60 ± 0.55a | 10.80 ± 1.66a |
| K [mg 100 g^-1^] | 23.23 ± 2.16a | 20.65 ± 2.58a | 24.49 ± 1.32a | 24.89 ± 0.69a |
| Rhizosphere pH | 6.73 ± 0.03b | 6.81 ± 0.04b | 6.13 ± 0.03c | 7.12 ± 0.14a |
| Moisture content [%] | 18.91 ± 0.01b | 23.15 ± 0.0a | 19.78 ± 0.01ab | 19.87 ± 0.03ab |

**Supplementary Figures**

**
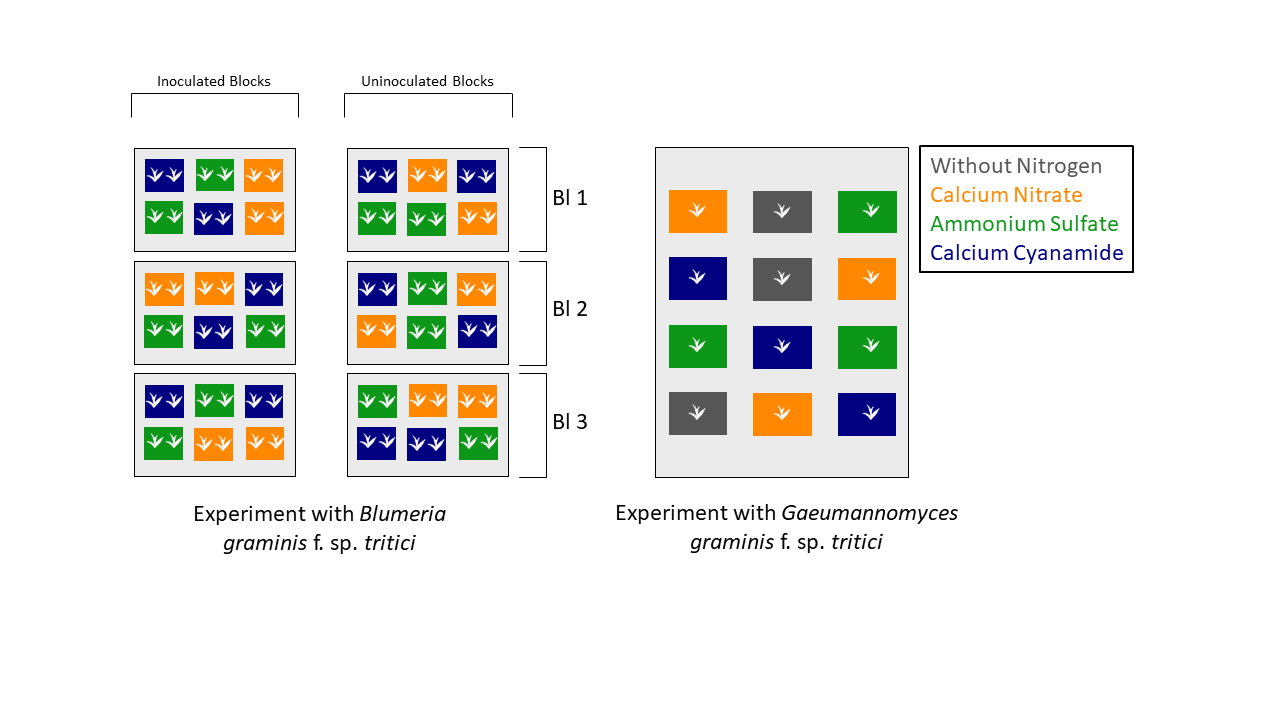
**

**A**

**B**

**Supplementary Figure 1** | Experimental Setup of the Experiment with (A) *Blumeria* *graminis* f. sp. *tritici* and (B) *Gaeumannomyces graminis* f.sp. *tritici.*

**A**

**B
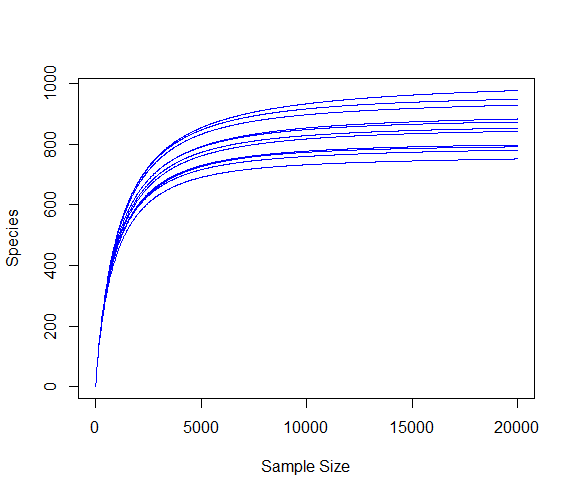
**

**
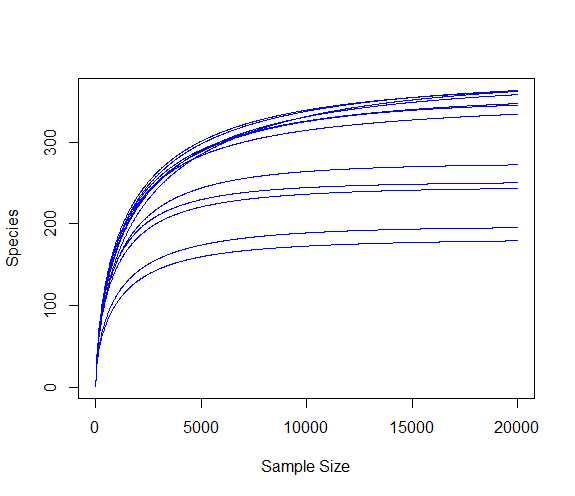
**

**Supplementary Figure 2** | Rarefaction curves for the (A) bacterial and (B) fungal dataset.


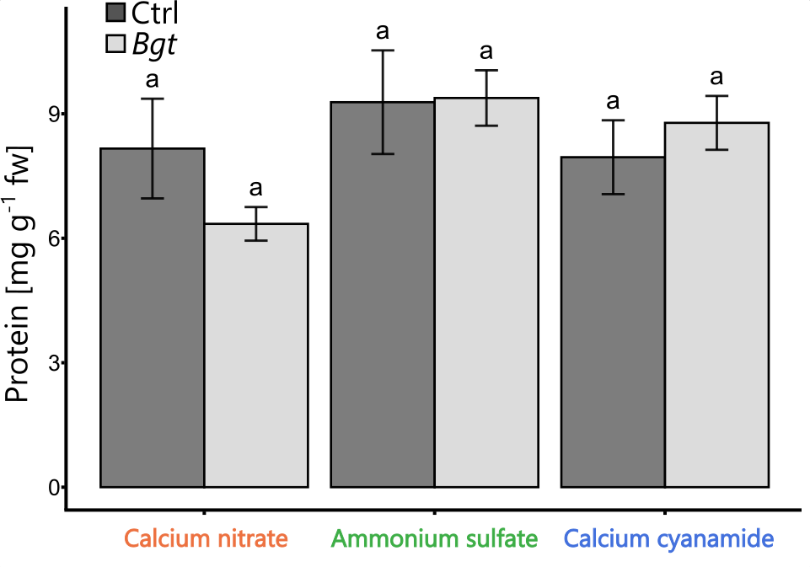


**A**


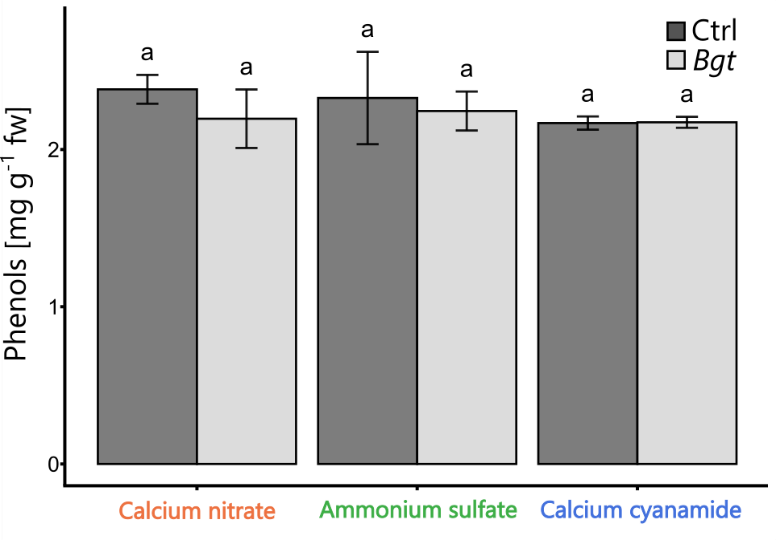


**B**

**Supplementary Figure 3** | (A) Protein [mg g^-1^ fw] and (B) phenols [mg g^-1^ fw] of winter wheat plants after 28 dpi treated with different nitrogen applications (calcium nitrate, ammonium sulfate, calcium cyanamide) under control conditions and *Bgt* infection. Data represent mean values of three biological replicates ± SE. The different lowercase letters indicate a significant difference at 0.05 probability level according to Tukey multiple comparison test.


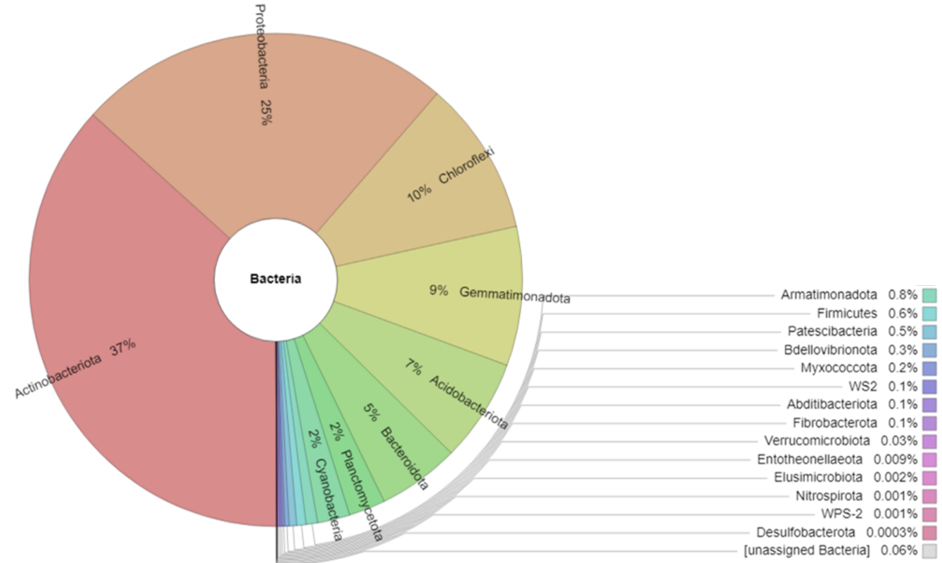


**Supplementary Figure 4** | Relative abundances of the phyla detected within the bacterial dataset.


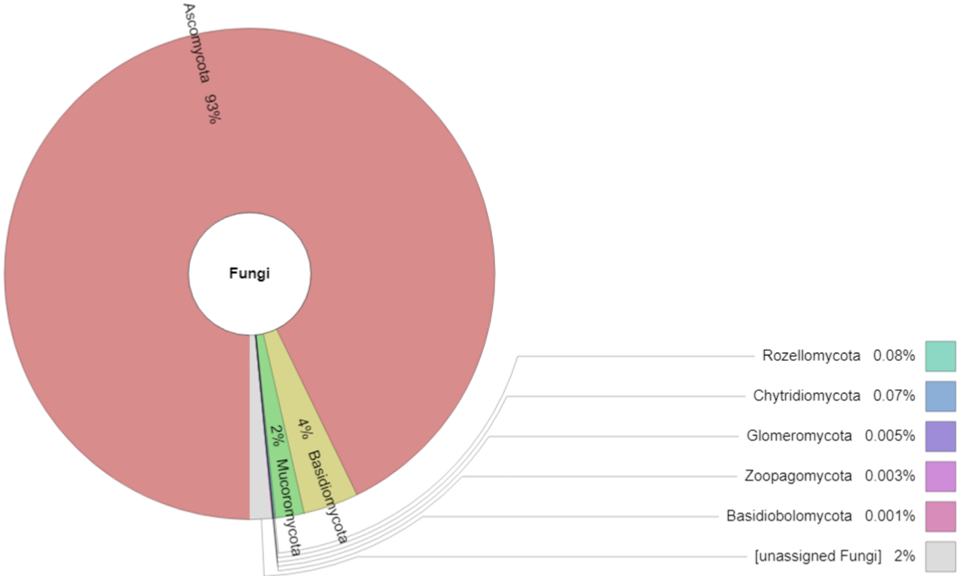


**Supplementary Figure 5** | Relative abundances of the phyla detected within the fungal dataset.


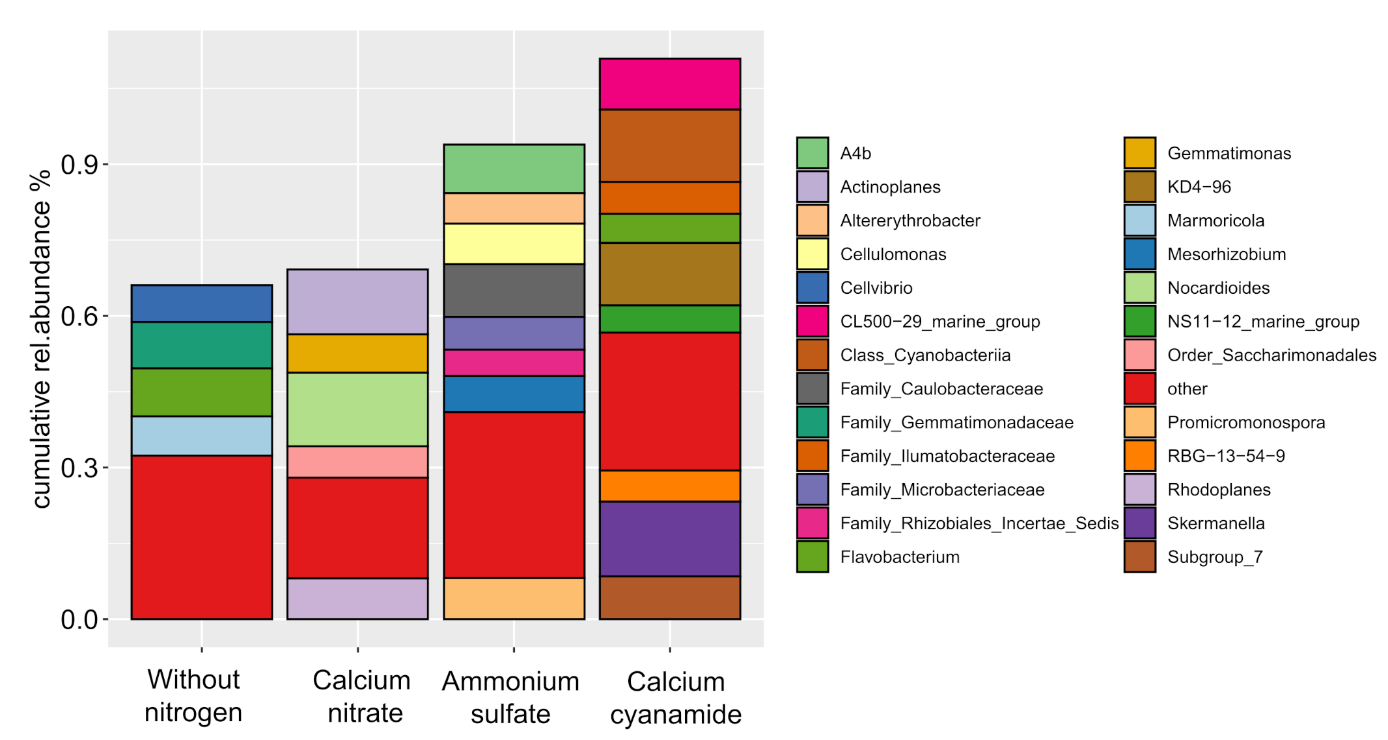


**Supplementary Figure 6 | Relative abundances of bacterial ASVs that are unique for each fertilization treatment clustered to the genus level (in case of ASVs that were not characterized at the genus level, their closest taxonomical level identified is reported). Only the taxonomic clades that account for abundance >0.05% are displayed.**


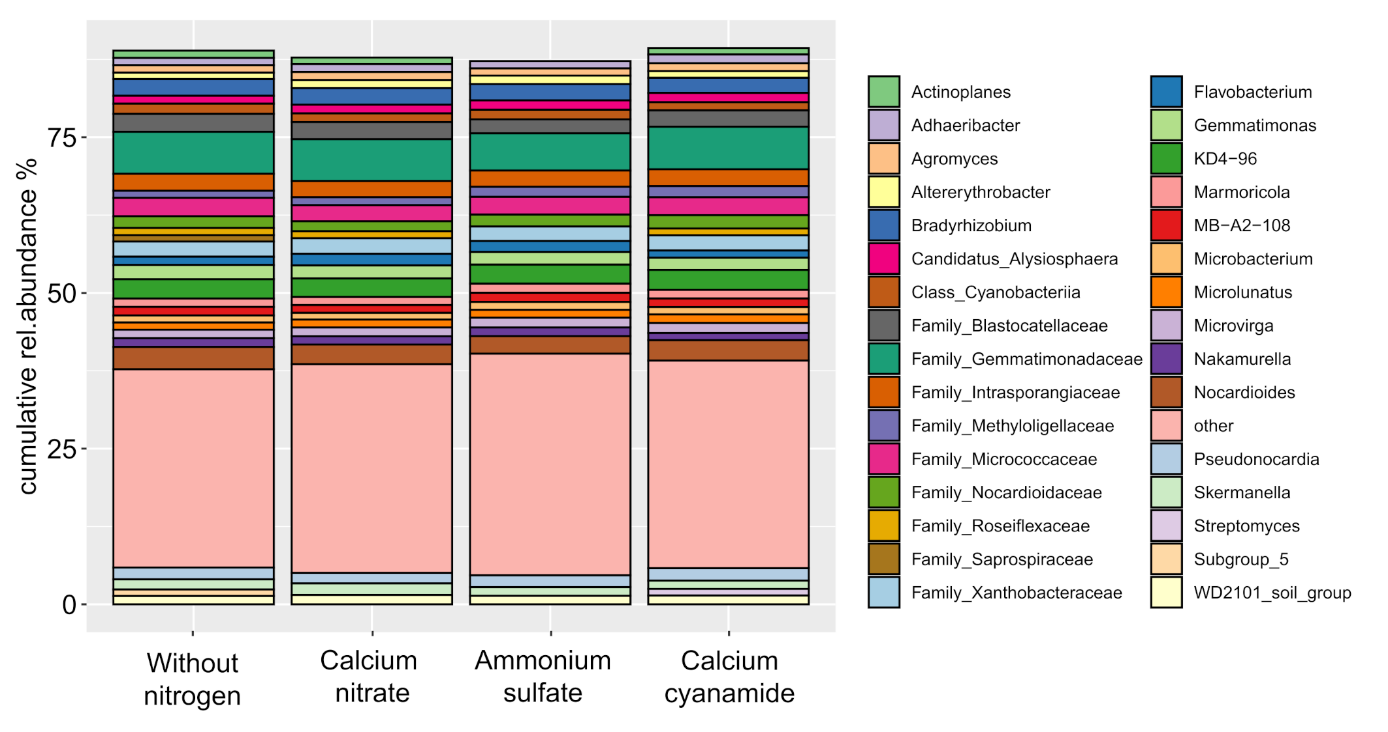


**Supplementary Figure 7 | Relative abundances of bacterial ASVs that are shared across each fertilization treatment clustered to the genus level (in case of ASVs that were not characterized at the genus level, their closest taxonomical level identified is reported). Only the taxonomic clades that account for abundance >1% are displayed.**


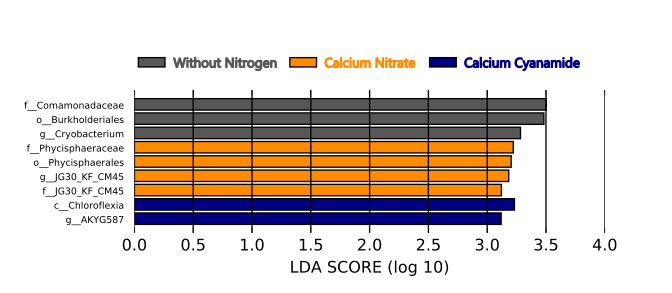


**Supplementary Figure 8 |** Linear discriminant analysis (LDA) scores computed for bacterial features differentially abundant between the different fertilization treatments.


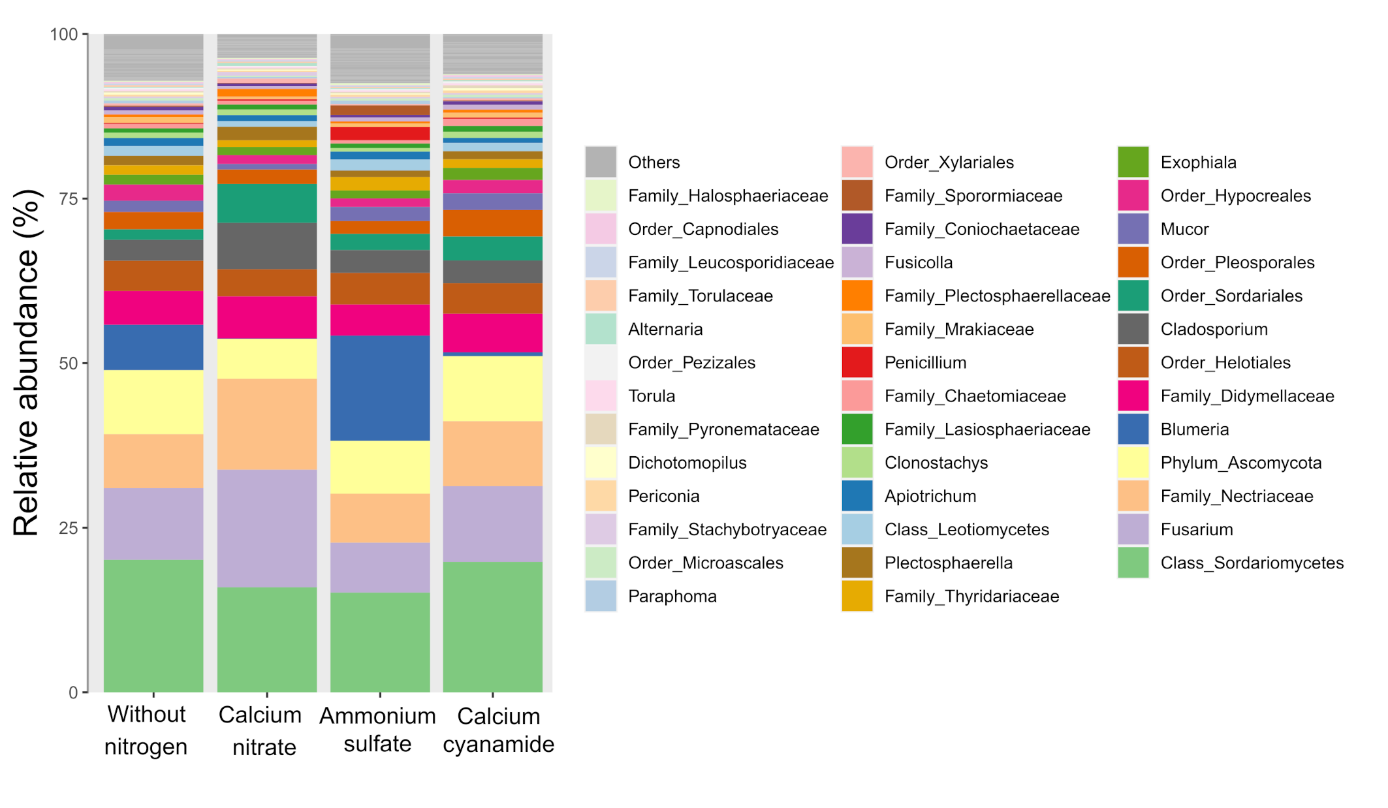


**Supplementary Figure 9 | Relative abundances of fungal ASVs clustered to the genus level (in case of ASVs that were not characterized at the genus level, their closest taxonomical level identified is reported).**


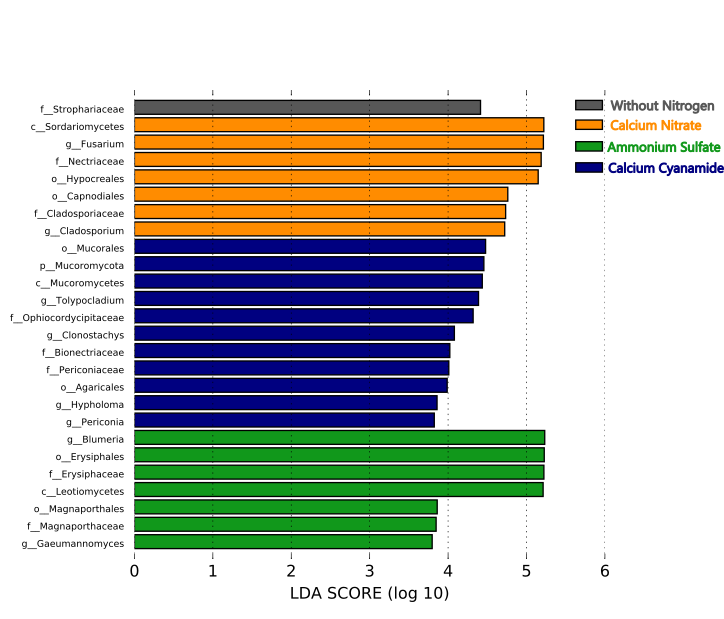
**Supplementary Figure 10 |** Linear discriminant analysis (LDA) scores computed for fungal features differentially abundant between the different fertilization treatments.
